# Disarib, a Specific BCL2 Inhibitor, Induces Apoptosis in Triple-Negative Breast Cancer Cells and Impedes Tumour Progression in Xenografts by Altering Mitochondria-Associated Processes

**DOI:** 10.3390/ijms25126485

**Published:** 2024-06-12

**Authors:** Meghana Manjunath, Febina Ravindran, Shivangi Sharma, Humaira Siddiqua, Sathees C. Raghavan, Bibha Choudhary

**Affiliations:** 1 Department of Biotechnology and Applied Bioinformatics, Institute of Bioinformatics and Applied Biotechnology, Electronic City Phase 1, Bengaluru 560100, India; 2 Indian Institute of Science, Bengaluru 560012, India; humairas@iisc.ac.in (H.S.); sathees@iisc.ac.in (S.C.R.)

**Keywords:** triple-negative breast cancer, BCL2 inhibitors, gene expression, energy metabolism

## Abstract

Targeted cancer therapy aims to disrupt the functions of proteins that regulate cancer progression, mainly by using small molecule inhibitors (SMIs). SMIs exert their effect by modulating signalling pathways, organelle integrity, chromatin components, and several biosynthetic processes essential for cell division and survival. Antiapoptotic protein BCL2 is highly upregulated in many cancers compared with normal cells, making it an ideal target for cancer therapy. Around 75% of primary breast cancers overexpress *BCL2*, providing an opportunity to explore BCL2 inhibitors as a therapeutic option. Disarib is an SMI that has been developed as a selective BCL2 inhibitor. Disarib works by disrupting BCL2-BAK interaction and activating intrinsic apoptotic pathways in leukemic cells while sparing normal cells. We investigated the effects of Disarib, a BCL2 specific inhibitor, on breast cancer cells and xenografts. Cytotoxicity and fluorometric assays revealed that Disarib induced cell death by increasing reactive oxygen species and activating intrinsic apoptotic pathways in Triple-Negative Breast Cancer cells (MDA-MB-231 and MDA-MB-468). Disarib also affected the colony-forming properties of these cells. MDA-MB-231- and MDA-MB-468-derived xenografts showed a significant reduction in tumours upon Disarib treatment. Through the transcriptomics approach, we also explored the influence of BCL2 inhibitors on energy metabolism, mitochondrial dynamics, and epithelial-to-mesenchymal transition (EMT). Mitochondrial dynamics and glucose metabolism mainly regulate energy metabolism. The change in energetics regulates tumour growth through epithelial–mesenchymal transition, and angiogenesis. RNA sequencing (RNAseq) analysis revealed that BCL2 inhibitors ABT-199 and Disarib maintain Oxphos levels in MDA-MB-231. However, key glycolytic genes were significantly downregulated. Mitochondrial fission genes were seen to be downregulated both in RNAseq data and semi quantitative real time polymerase chain reaction (qRTPCR) in Disarib-treated TNBC cells and xenografts. Lastly, Disarib inhibited wound healing and epithelial-to-mesenchymal transition. This study showed that Disarib disrupts mitochondrial function, activates the intrinsic apoptotic pathway in breast cancer, and inhibits epithelial-to-mesenchymal transition both in vitro and in vivo. These findings highlight Disarib’s potential as a multifaceted therapeutic strategy for patients with Triple-Negative Breast Cancer.

## 1. Introduction

Invasive breast cancer affects one in nine women [1]. Triple-Negative Breast Cancer (TNBC) is one invasive cancer that grows rapidly and metastasises [2]. One of the reasons for metastasis is the activation of the epithelial-to-mesenchymal transition (EMT) programme, which gives migratory potential to the cancer cells. EMT is a process where cells lose tight and adherent junctions and simultaneously gain mesenchymal characteristics [3]. EMT is regulated by hypoxia, growth factor signalling, and stroma-tumour interactions [4]. A crosstalk exists between the transcription factors, feedback loops, and EMT-inducing signals to initiate the EMT programme [5]. Transcription factors TWIST, SNAIL, SLUG, ZEB 1 and 2, and SERPINE participate in the EMT process. In TNBC cells, mesenchymal markers and EMT transcription factors are overexpressed. ZEB1 overexpression in TNBC activates EMT and is correlated with poor prognosis [6]. Overexpression of vimentin, CD44, gives metastatic potential to TNBC cells [7]. 

One of the significant factors associated with EMT and leading to metastasis is metabolic reprogramming in TNBC cells [8]. One of the features of cancer cells is that they shift to glycolysis from oxidative phosphorylation in aerobic conditions to rapidly generate the energy required for tumour progression and metastasis, which is called the Warburg effect [9]. Metabolic rewiring and the shift towards glycolysis trigger EMT-inducing transcription factors and promote metastasis and resistance [10]. Overexpression of glycolytic enzymes leads to the EMT process in breast cancer stem cells. TNBC cells possess a hybrid metabolic status having high glycolysis and a considerable level of oxidative phosphorylation (OXPHOS), including HIF-1/AMPK signalling [11]. Cells with this metabolic status show higher clonogenicity, proliferation rate, and metastatic potential. The hybrid metabolism provides plasticity to TNBC cells to conveniently switch between glycolysis and OXPHOS to combat drug action and modify the tumour environment [12].

Understanding tumour biology at the molecular level has given the prospect of specifically targeting deregulated signalling pathways and proteins. Targeted therapy has been designed against mutant kinases, tumour microenvironment, hormone and hormone receptors, etc., to lower toxicity than chemotherapy and radiotherapy [13]. Antiapoptotic protein BCL2 is highly upregulated in many cancers compared with normal cells, making it an ideal target for cancer therapy. BCL2 is highly expressed in approximately 75% of primary breast cancers [14]. Around 41% of Triple-Negative Breast Cancers and 19% of basal-like tumours show high BCL2 [15]. Analysis of the GENT2 database http://gent2.appex.kr/gent2/ (accessed on 1 April 2024) further supports this trend, revealing that 9.2% of patients with TNBC display BCL2 overexpression [16]. In contrast, the luminal subtype, primarily targeted by endocrine therapy because of the presence of oestrogen receptor (ER), progesterone receptor (PR), and human epidermal growth factor receptor 2 (HER2) expression, exhibits a predominantly medium BCL2 expression profile, with only 3% of patients showing high BCL2 levels. This differential BCL2 expression pattern underscores the ineffectiveness of endocrine therapy in TNBC [17]. Chemotherapy remains the mainstay of treatment for TNBC, but disease recurrence remains a significant challenge because of the inherent aggressiveness of the cancer. Given the high prevalence of BCL2 overexpression in TNBC, targeting this anti-apoptotic protein with specific inhibitors emerges as a promising therapeutic strategy for this challenging malignancy.

BCL2 heterodimerises with BAK/BAX and is essential for maintaining mitochondrial outer membrane integrity. Many small-molecule inhibitors have been developed that break the interaction between BCL2 (antiapoptotic) and its partner BAK/BAX (proapoptotic). Gossypol, AT101, Obatoclax, ABT-737, ABT-263, Tw37, YC137, and HA14-1 are some of the molecules synthesised that are pan-active [18]. ABT-199 is BCL2-specific and FDA-approved for leukaemia and lymphoma [19]. All these are BCL2 homology domain 3 (BH3) mimetics. Another small molecule inhibitor, Disarib, has been developed as a selective BCL2 inhibitor. Z24 is one of the molecules belonging to the 3-substituted indolin-2-ones class of compounds having anti-cancer and anti-angiogenic properties, that could block BCL2 based on in silico analysis [20]. Using backbones derived from Z24, a series of 17 derivatives were synthesised. Among the 17 derivatives, Disarib exhibited a favourable binding energy of −10.4 kcal·mol^−1^ through docking studies and was further characterised and assessed for its anticancer properties [21]. Target specificity was confirmed using BCL2 knockdown cells, which offered resistance to Disarib. Biochemical, biophysical, *and* in silico studies showed a high affinity of Disarib to BCL2 but not to BCL-xL, BCL2A1, etc. There was a 67-fold reduced BCL2–Disarib interaction in BH1 domain-deleted mutants, suggesting a strong involvement of the BH1 domain in binding [21]. Disarib disrupts the BCL2-BAK interaction while sparing BCL2-BAX, activating an intrinsic apoptotic pathway in leukemic cells. Disarib caused significant tumour reduction in mouse allograft and xenograft models and displayed platelet-sparing properties without causing substantial side effects [22]. Disarib, compared with ABT-199, exhibited better efficacy in cell lines and mouse models. When Disarib was administered orally, dose-dependent regression in Ehrlich ascites carcinoma (EAC) and Dalton’s lymphoma (DLA) tumours in syngeneic mouse models was observed. Toxicity (single dose) studies of Disarib in male and female normal mice revealed no changes in the treatment group compared to controls [23]. 

This study investigates the potential of Disarib, a small molecule inhibitor of the antiapoptotic protein BCL2, as a therapeutic strategy for Triple-Negative Breast Cancer. This study examines how Disarib disrupts BCL2 function, activates apoptosis, and disrupts mitochondrial function, cellular energetics, and epithelial-to-mesenchymal transition, a process linked to metastasis, in TNBC cells and xenograft models. 

## 2. Results

### 2.1. Disarib Induces Cytotoxicity in TNBC Cell Lines in a Concentration-Dependent Manner without Causing Cell Cycle Arrest

**Figure 1:**
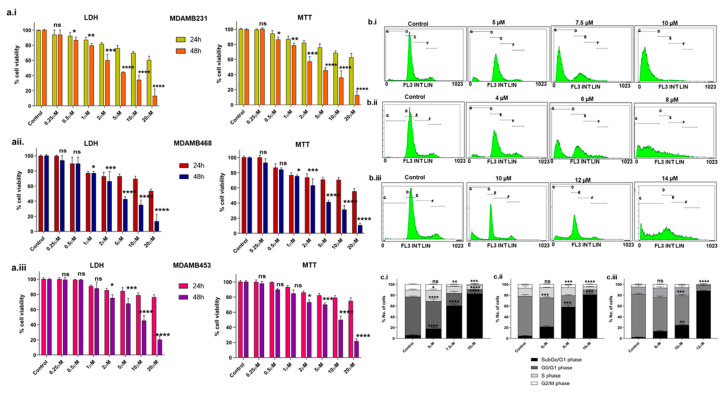
Evaluation of the effect of Disarib on TNBC cell lines. The cells were seeded in 96-well plates, incubated for 24 h, and treated with increasing concentrations of Disarib (0.25 µM, 0.5 µM, 1 µM, 2 µM, 5 µM, 10 µM, 20 µM) for MTT and LDH assays for 24 and 48 h. Cells treated with DMSO were considered as vehicle control. (**a.i**) LDH and MTT assay for Disarib-treated MDA-MB-231; (**a.ii**) LDH and MTT assay for Disarib-treated MDA-MB-468; and (**a.iii**) LDH and MTT assay for Disarib-treated MDA-MB-453. Effect of Disarib cell cycle phase distribution on (**b.i**) MDA-MB-231, (**b.ii**) MDA-MB-468, and (**b.iii**) MDA-MB-453. Cells were seeded in a 6-well plate and treated with Disarib for 48 h. After the 48 h incubation, cell cycle distribution was quantified and analysed using a flow cytometer. The bar diagram shows the percentage of cells distributed in each cell cycle phase. Each experiment was repeated a minimum of three times and plotted as bar graphs with error bars for (**c.i**) MDA-MB-231, (**c.ii**) MDA-MB-468, and (**c.iii**) MDA-MB-453. The *p*-value was calculated between the control and Disarib groups, where ns: non-significant, *p* < 0.05 = *, *p* < 0.01 = **, *p* < 0.001 = ***, *p* < 0.0001 = ****.

We examined the levels of BCL2 in the following three TNBC cell lines: MDA-MB-231, MDA-MB-468, and MDA-MB-453. The results suggest that MDA-MB-231 has higher BCL2, followed by MDA-MB-468 and MDA-MB-453 (Supplementary Figure S1a.i,a.ii). The cytotoxic effects of Disarib on MDA-MB-231, MDA-MB-468, and MDA-MB-453 were assessed by performing 3-(4,5-dimethylthiazol-2-yl)-2,5-diphenyltetrazolium bromide (MTT) and Lactate Dehydrogenase (LDH) assays (Figure 1a.i–a.iii). The cells were treated with varying concentrations (0.25 µM to 20 µM) of Disarib and incubated for 24h and 48 h. Post incubation, MTT and LDH assays were conducted. Cells treated with dimethyl sulfoxide (DMSO) were used as vehicle control. At 24 h, no significant cell death was seen upon Disarib treatment in any of the three cell lines. At 48 h, Disarib induced cytotoxicity in a concentration-dependent manner in all three cell lines. In MDA-MB-231, 50% cell death was seen at 4.5 µM, 4.3 µM for MDA-MB-468 and ~7.7 µM for MDA-MB-453 at 48 h. Both MTT and LDH showed comparable results.

Propidium iodide (PI) staining and flow cytometric analysis revealed a typical cell cycle distribution with G1, S, and G2/M phases in control cells treated with DMSO for all three lines including MDA-MB-231, MDA-MB-468, and MDA-MB-453 (Figure 1b.i–b.iii). A slight increase in the subG0/G1 population, indicative of cell death, was observed at IC_50_ Disarib concentrations (5 µM for MDA-MB-231, 4 µM for MDA-MB-468). At higher concentrations of double IC_50_ (10 µM for MDA-MB-231, 8 µM for MDA-MB-468, 14 µM for MDA-MB-453), a significant increase in subG0/G1 was observed in all three cell lines (Figure 1c.i–c.iii), suggesting Disarib primarily induces cell death rather than cell cycle arrest after 48 h of treatment.

### 2.2. Disarib Induces Mitochondria-Mediated Apoptotic Cell Death in MDA-MB-231, MDA-MB-468, and MDA-MB-453

**Figure 2:**
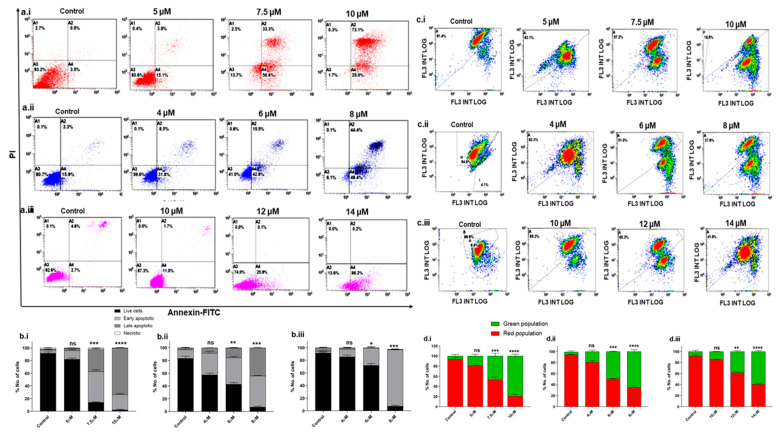
Annexin-FITC/PI staining to examine the level of apoptosis in TNBC cells following Disarib treatment. (**a.i**) MDA-MB-231, (**a.ii**) MDA-MB-468, and (**a.iii**) MDA-MB-453 cells were treated with Disarib for 48 h and were stained with Annexin V-FITC/PI. The samples were analysed with flow cytometry. The results are represented as a dot plot with the lower left quadrant showing cells that are Annexin-V (-) PI (-), the lower right quadrant showing early apoptotic cells stained with Annexin-V only, the upper right showing Annexin-V (+) PI (+) cells that are late-apoptotic, and the upper left showing dead cells with Annexin-V (-) PI (+). Each experiment was repeated a minimum of 3 times and plotted as bar graphs with error bars for (**b.i**) MDA-MB-231 (**b.ii**), MDA-MB-468, and (**b.iii**) MDAM453. The effect of Disarib on mitochondrial membrane potential in MDAMB cell lines was investigated by JC1 staining. A fluorescence shift from red to green as a result of Disarib is shown as dot plots, and the percentage of the population of cells is plotted as a bar graph. (**c.i**,**d.i**) MDA-MB-231 (**c.ii**,**d.ii**) MDA-MB-468 (**c.iii**,**d.iii**) MDA-MB-453. Each experiment was repeated for a minimum of three times, and the *p*-value was calculated between the control and Disarib-treated cells, where ns: non-significant *p* < 0.05 = *, *p* < 0.01 = **, *p* < 0.001 = ***, *p* < 0.0001 = ****.

Once it was established that Disarib induces cytotoxicity in TNBC cell lines, we investigated the mode of cell death further. Cell death could be through apoptosis or necrosis. The Annexin-V/PI double staining method was employed to understand the mode of cell death. TNBC cell lines were treated with different concentrations of Disarib as follows: 5, 7.5, and 10 µM for MDA-MB-231, 4, 6, and 8 µM for MDA-MB-468 and 10, 12, and 14 µM for MDA-MB-453 for 48 h and then stained with Annexin V-FITC/PI. DMSO-treated cells were used as vehicle control. The overall results suggest that the apoptotic cell fraction increased in a concentration-dependent manner in all three cell lines. In MDA-MB-231, at the 5 µM Disarib concentration, ~73% of the cells were at the early apoptotic stage, and 33% of the cells were at the late apoptotic stages; at the highest concentration (10 µM), 73% of the cells had reached the late apoptotic stage (Figure 2a.i). In MDA-MB-468, the highest concentration (8 µM) showed ~48% early and 44% late apoptotic cells (Figure 2a.ii). On the other hand, ~76% of the MDA-MB-453 cells were seen in the early apoptotic stages at the 14 µM concentration of Disarib (Figure 2a.iii). MDA-MB-453 did not show an apoptotic cell fraction at the lower drug concentration, possibly because of lower sensitivity to Disarib than MDA-MB-231 and MDA-MB-468. Among the three cell lines, MDA-MB-231 showed more sensitivity to Disarib-induced apoptotic cell death than MDA-MB-468 and MDA-MB-453. Also, the necrotic fractions in all the cell lines were negligible, indicating that Disarib induces apoptosis in MDA-MB-231, MDA-MB-468, and MDA-MB-453 cell lines (Figure 2b.i–b.iii).

Since Disarib targets mitochondrial membrane proteins, we studied the effect of Disarib on mitochondrial membrane potential. The MDA-MB-231 (5, 7.5, and 10 µM), MDA-MB-468 (4, 6 and 8 µM), and MDA-MB-453 (10, 12 and 14 µM) cell lines were treated with different concentrations of Disarib, incubated for 48 h, and stained with JC1 dye. The control cells with DMSO showed mostly red fluorescence in all three cell lines, indicating healthy mitochondria. Upon Disarib treatment, a shift in red fluorescence to green was observed in all three cell lines. MDA-MB-231 (Figure 2c.i,d.i) displayed an 81% shift at the highest concentration, MDA-MB-468 (Figure 2c.ii,d.ii) showed a 63% shift, and MDA-MB-453 (Figure 2c.iii,d.iii) showed a 58% shift from the red to green population. A disruption in the mitochondrial membrane potential in all three cell lines upon Disarib treatment in a concentration-dependent manner was observed. These results showed the loss of mitochondrial membrane potential and possible activation of the intrinsic apoptotic pathway.

The level of proteins associated with the mitochondria-mediated intrinsic apoptotic pathway was also investigated. Cell lysates were prepared from MDA-MB-231 and MDA-MB-468 cells. DMSO-treated cells were used as a control. For MDA-MB-231, three concentrations of Disarib were used, i.e., 2.5, 5, and 7 µM, and for MDA-MB-468, 2, 4, and 6 µM Disarib were used (Supplementary file S2). Interestingly, a significant increase in the expression of proapoptotic proteins and a reduction in the antiapoptotic proteins was observed at higher concentrations of Disarib. Compared with the control, the antiapoptotic protein BCL2 was downregulated after 48 h of treatment in the treated samples. In MDA-MB-231, maximal downregulation of BCL2 was observed at 7.5µM, whereas this was observed at 6µM in MDA-MB-468 cells. We also examined the levels of proapoptotic proteins such as BAK, cytochrome C, and APAF-1. The proapoptotic proteins were upregulated in MDA-MB-231 and MDA-MB-468 (Supplementary Figure S2a,b). Caspase 3 showed downregulation in its precursor forms in MDA-MB-231 and MDA-MB-468 treated with Disarib. The downregulation of BCL2 and upregulation of the apoptosome complex were observed in both TNBC cell lines, indicating activation of the intrinsic pathway of apoptosis upon Disarib treatment.

### 2.3. Disarib Significantly Reduces the Colony-Forming Ability of TNBC Cell Lines

**Figure 3:**
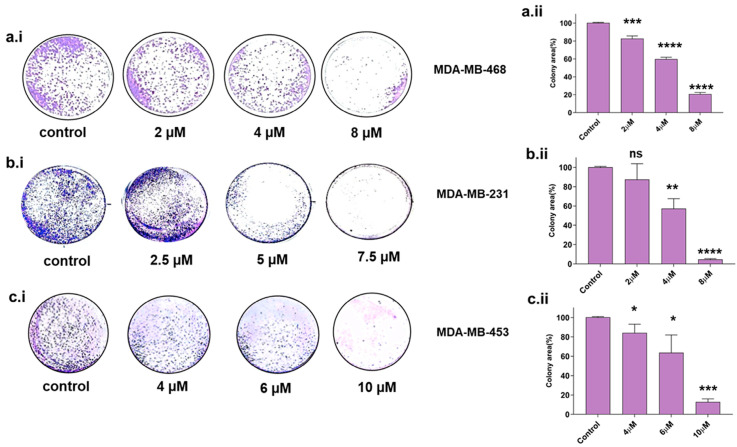
Colony-forming assay in Disarib-treated (**a.i**) MDA-MB-231, (**b.i**) MDA-MB-468, and (**c.i**) MDA-MB-453. The colony number decreases with increasing concentration of Disarib in all three cell lines. The bar graph depicts the quantification of the per cent of stained cells in an area for (**a.ii**) MDA-MB-231, (**b.ii**) MDA-MB-468, and (**c.ii**) MDA-MB-453, where ns: non-significant, *p* < 0.05 = *, *p* < 0.01 = **, *p* < 0.001 = ***, *p* < 0.0001 = ****.

The colony-forming property is a sign of undifferentiated cancer stem cells [24,25]. Therefore, we assessed the colony-forming ability in TNBC cell lines upon Disarib treatment. Cells were seeded at a lower density in a 12-well plate, treated with different concentrations of Disarib, and observed for colony formation after 10 days. The colonies were stained with crystal violet, and the colony area was measured using Image J software version 1.52a, (https://imagej.nih.gov/ij/ accessed on 5 March 2024). For MDA-MB-231, 2, 4, and 8 µM of Disarib was used for the assay. We observed a reduction in colony formation in a concentration-dependent manner in MDA-MB-231. Disarib at a concentration of 5 µM significantly reduced colonies in MDA-MB-231 cells (Figure 3b.i). A maximum of 90% reduction in the colonies was seen at the highest concentration (8 µM) compared with the control in MDA-MB-231 (Figure 3b.ii). In MDA-MB-468, a concentration range of 2–8 µM was used for the assay. MDA-MB-468 showed a reduction in colony formation at a starting concentration of 2 µM, and a further reduction in colonies was observed as the drug concentration increased (Figure 3a.i). Disarib at 8 µM showed around an 80% decrease in colony formation compared with the control (Figure 3a.ii). However, in MDA-MB-453, 4 and 6 µM displayed a 30–40% (Figure 3c.i) reduction, and the highest concentration (10 µM) showed around an 80% reduction in the colonies compared with the control (Figure 3c.ii).

### 2.4. Disarib Induces Reactive Oxygen Species in MDA-MB-231, MDA-MB-468, and MDA-MB-453

**Figure 4:**
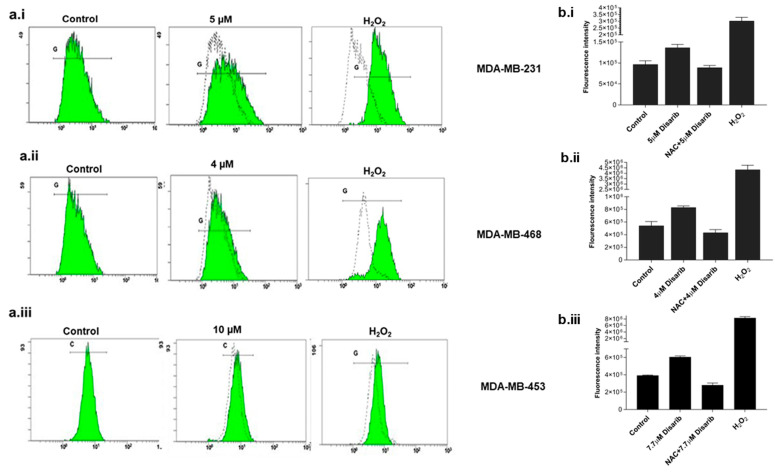
Effect of Disarib on reactive oxygen species (ROS). A spectrophotometric measure of fluorescence intensity by H2DCFDA staining to assess ROS. The graph was plotted against the fluorescence intensity. Here, 20 mM N-acetyl cysteine (NAC) was used as a ROS scavenger, and treatment with H_2_O_2_ served as the positive control. (**a.i**) MDA-MB-231, (**a.ii**) MDA-MB-468, and (**a.iii**) MDA-MB-453. A flow cytometric analysis of ROS using H2DCFDA staining. A shift in the histogram was observed compared with the control histogram upon Disarib treatment in (**b.i**) MDA-MB-231, (**b.ii**), MDA-MB-468, and (**b.iii**) MDA-MB-453. Each experiment was repeated a minimum of 3 times, and the *p*-value was calculated between the control and Disarib treatment, where *p* < 0.05 = *, *p* < 0.01 = **.

We examined reactive oxygen species (ROS) levels to check whether the cytotoxicity observed was due to ROS production. BCL2 is an essential component of the mitochondrial outer membrane; any effect on BCL2 would alter mitochondrial function and ROS generation. Many drugs generate ROS to trigger mitochondrial damage. To investigate the ROS generation by Disarib in TNBC cells, fluorogenic dye H2DCFDA (2′,7′–dichlorofluorescein diacetate) was used. All three cell lines were treated with an IC_50_ dose for different time points of 15 min, 30 min, and 1, 2, 4, 24, and 48 h, and the ROS was measured spectrophotometrically. Maximum ROS was generated at 2 h in MDA-MB-231, at 30 min in MDA-MB-468, and 4 h in MDA-MB-453. Further, with the addition of a ROS scavenger, N-acetyl-l-cysteine (NAC, 20 mM), ROS generation halted in all three cell lines treated with Disarib (Figure 4b.i–b.iii). Cells treated with H_2_O_2_ were used as a positive control. To further confirm Disarib-induced ROS, MDA-MB-231 cells were treated with Disarib for 2 h, MDA-MB-468 for 30 min, and MDA-MB-453 for 4 h and then stained with H2DCFDA, and the fluorescence was measured in a flow cytometer. A shift in the fluorescence histogram was observed compared to the control (measured by median fluorescence intensity), which was noted as a measure of intracellular ROS. In MDA-MB-231 cells, 5 µM Disarib induced ROS (Figure 4a.i). In MDA-MB-468, a shift in the histogram at 30 min was observed with 4 µM Disarib (Figure 4a.ii). In MDA-MB-453, at 4 h, ROS were induced by 7.5 µM Disarib (Figure 4a.iii). Cells treated with H_2_O_2_ were used as a positive control. These results suggest ROS production upon Disarib treatment in all three cell lines.

### 2.5. Disarib Inhibits Tumour Growth in Human Triple-Negative Breast Cancer Cell Line-Derived Xenograft Models

**Figure 5:**
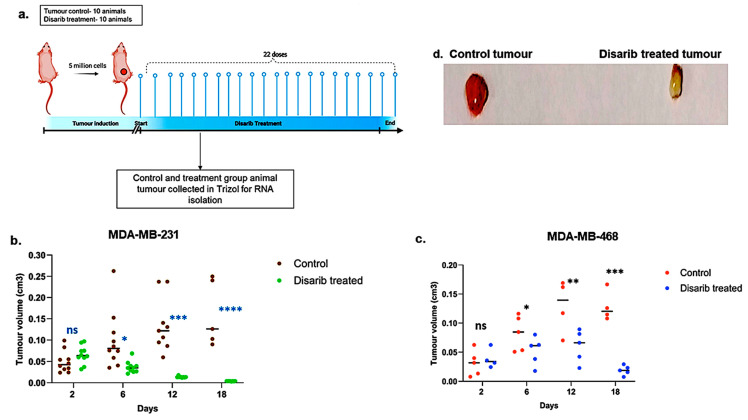
Effect of Disarib on tumour proliferation in cell line-derived xenografts. (**a**) Methodology opted for the experiment. Animals were treated with Disarib (50 mg/kg, 22 doses, daily, n = 10), and tumour regression was compared with the control group. (**b**) Graph showing tumour regression in MDA-MB-231-injected nude mice after Disarib treatment. (**c**) Graph showing tumour regression in MDA-MB-468-injected nude mice after Disarib treatment. Tumour volume is measured in cm^3^ for control and treated animals as plotted. where ns: non-significant *p* < 0.05 = *, *p* < 0.01 = **, *p* < 0.001 = ***, *p* < 0.0001 = ****. (**d**) Representative image of a cell line-derived tumour control and a tumour treated with Disarib.

Disarib has shown tumour regression in vivo in the EAC breast cancer model when given intraperitoneally and orally. This study was approved by the animal ethics committee at the Institute of Bioinformatics and Applied Biotechnology (IBAB) under protocol number IAEC/IBAB/08/12/2019. Here, we assessed the efficacy of Disarib in cell line-derived xenografts. The TNBC cell lines MDA-MB-231 and MDA-MB-468 were used for this study. Swiss albino nude mice were subcutaneously injected with 5 × 10^6^ MDA-MB-231/MDA-MB-468 cells per animal mixed with matrigel. The animals were divided into control and treatment groups of ten. Once the tumour volume reached around 20 mm^3^, the animals in the treatment group received 50 mg/kg body weight of Disarib orally for 22 days (Figure 5a). Tumour volume was measured every other day for mice bearing MDA-MB-231 and MDA-MB-468 xenografts. As depicted in Figure 5b,c, tumour volumes in both Disarib-treated groups (MDA-MB-231 and MDA-MB-468) were comparable to control groups on day 2. However, from day six onwards, a significant divergence was observed. Control animals exhibited progressive tumour growth, whereas Disarib treatment resulted in a marked reduction in tumour volume for both xenograft models (MDA-MB-231 and MDA-MB-468) compared with their respective controls (Figure 5d). These findings demonstrate Disarib’s efficacy in impeding tumour progression in vivo, further highlighting its potential as a therapeutic strategy for TNBC.

### 2.6. Disarib Significantly Reduced Glycolysis and Caused a Mild Decrease in Oxidative Phosphorylation in TNBC Cell Lines

**Figure 6:**
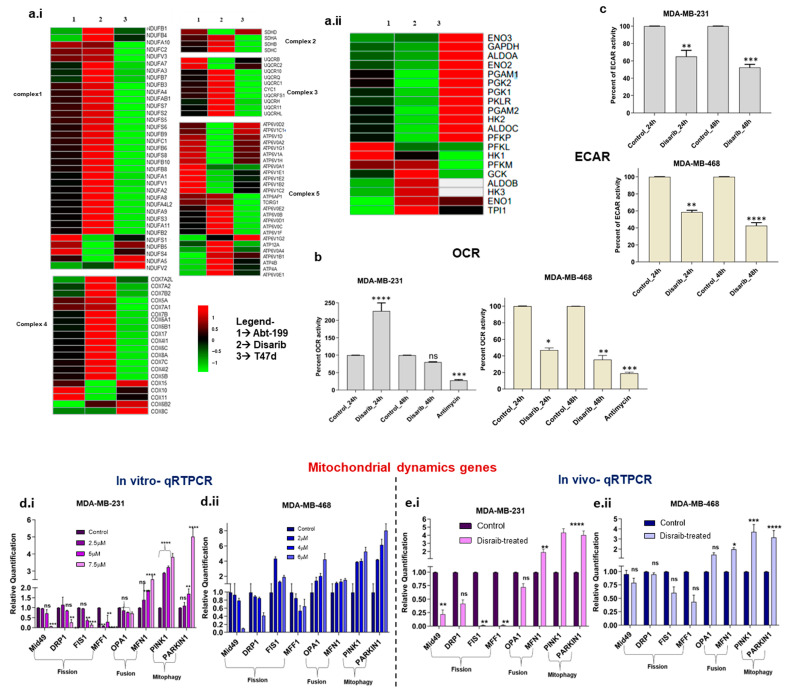
Heatmaps depicting gene expression changes in Disarib- and ABT199-treated MDA-MB-231 (BCL2 high) and Disarib inhibitor-treated T47D (BCL2 negligible) for (**a.i**) genes belonging to 5 complexes of OXPHOS and (**a.ii**) genes from the glycolysis pathway. The red colour represents upregulation, and the green colour represents downregulation. (**b**)**.** Extracellular acidification rate (ECAR) assay for Disarib-treated MDA-MB-231 and MDA-MB-468. (**c**)**.** Oxygen consumption rate (OCR) assay for Disarib-treated MDA-MB-231 and MDA-MB-468. (**d.i**) qRTPCR of fission, fusion, and mitophagy markers for Disarib-treated MDA-MB-231. (**d.ii**) qRTPCR of fission, fusion, and mitophagy markers for Disarib-treated MDAM468. (**e.i**) qRTPCR of fission, fusion, and mitophagy markers for Disarib-treated MDA-MB-231 xenograft. (**e.ii**) qRTPCR of fission, fusion, and mitophagy markers for Disarib-treated MDAM468 xenograft. The dark purple and blue bars represent control samples, and the light purple and blue bars represent Disarib-treated samples. where ns: non-significant *p* < 0.05 = *, *p* < 0.01 = **, *p* < 0.001 = ***, *p* < 0.0001 = ****.

Disarib, a BCL2 inhibitor, disrupted mitochondrial function by altering outer membrane potential. Therefore, we further investigated its effect on mitochondria-mediated processes, mitochondrial energy metabolism, and dynamics. Firstly, gene lists for glycolysis, oxidative phosphorylation (OXPHOS), fission, fusion, and mitophagy pathways were procured from the literature and the KEGG database. Secondly, log2fold changes in these genes were extracted from gene expression data of Disarib- and ABT-199-treated MDA-MB-231(TNBC) cells (Supplementary File S1). To isolate the effect of BCL2 on Disarib’s activity, we utilised T47D cells, a line previously characterised by our lab to have negligible BCL2 expression and minimal Disarib sensitivity [22]. This enabled a direct comparison of Disarib’s effects on BCL2-positive (MDA-MB-231) and BCL2-deficient (T47D) cells. While acknowledging potential inherent gene expression variations in these lines, our primary focus was on Disarib’s impact on cellular energy metabolism. By leveraging published data on the metabolic profiles of both MDA-MB-231 and T47D cells, we were able to analyse and interpret the observed changes in a more targeted manner [26,27]. A separate heatmap was plotted for the genes belonging to OXPHOS, glycolysis, fission and fusion, and mitophagy pathways. Regarding energy usage, the T47D cell line is glycolytic with negligible OXPHOS, whereas MDA-MB-231 cells are relatively highly glycolytic compared with MDA-MB-468. MDA-MB-468 has higher OXPHOS levels compared with MDA-MB-231. Nevertheless, both the cells show a hybrid metabolic phenotype MDA-MB-231, which has a hybrid energy metabolism exhibiting glycolysis and OXPHOS. Energy production is via Electron Transport Chain (ETC) complexes in mitochondria; we checked for any alteration in complexes 1–5 (Supplementary Figure S3a). There was no significant change in gene expression of the OXPHOS complexes in T47D cells. In contrast, there was a mild effect on MDA-MB-231 (Figure 6a.i). However, Disarib reduced the expression of key glycolytic enzymes ALDOA, HK2, PGAM1, and PGK2A in MDA-MB-231. The effect was not seen in T47D, indicating that any change in the energy metabolism pathway might be due to BCL2-specific inhibition (Figure 6a.ii). Disarib did not alter OXPHOS genes in T47D, indicating that the changes observed by all inhibitors are BCL2-specific. ABT-199-treated MDA-MB-231 showed a similar gene expression pattern to Disarib, where OXPHOS was unaltered, and a reduction in glycolysis was observed.

The functional impact of Disarib on energy metabolism was checked using an extracellular acidification assay (ECAR) and an oxygen consumption rate (OCR) assay. We performed a fluorometric extracellular acidification assay to measure the glycolytic activity of Disarib-treated MDA-MB-231 and MDA-MB-468 at 24 h and 48 h. A total of 80,000 cells were seeded for control, and Disarib treatment for both the cell lines and relative changes in lactic acid levels were normalised based on the protein quantification. The results suggested a 30% reduction in glycolysis at 24 h and a 50% reduction at 48 h in Disarib-treated MDA-MB-231 (Figure 6c). In MDA-MB-468, Disarib reduced glycolysis by 40% at 24 h and 60% at 48 h (Figure 6c). Further, we performed a fluorometric oxygen consumption rate assay to measure the oxidative phosphorylation activity of Disarib-treated MDA-MB-231 and MDA-MB-468 at 24 h and 48 h. A total of 80,000 cells were seeded for control and Disarib treatment in both cell lines. O_2_ was measured and normalised based on protein quantification. Antimycin A, a well-known OXPHOS inhibitor, was used as a control. The results suggested that in MDA-MB-231, Disarib-treated cells showed more OXPHOS than the controls at 24 h. However, at 48 h, there was a slight reduction in the levels of OXPHOS compared with the control (Figure 6b). Antimycin showed a 70% reduction in OXPHOS compared with the control in MDA-MB-231. In MDA-MB-468, 50% and 60% reductions in OXPHOS activity were observed at 24 h and 48 h, respectively (Figure 6b). Antimycin showed an 80% reduction in OXPHOS activity in MDA-MB-468.

Disarib did not reduce OXPHOS significantly, so we used Metformin, a well-known complex I inhibitor, in combination with Disarib. Cancer cells resist drugs by modifying their energy metabolism. Therefore, we aimed to block both metabolic pathways. Disarib treatment did not significantly suppress OXPHOS relative to glycolysis in MDA-MB-231 cells. Conversely, MDA-MB-468 cells, which exhibit a higher dependence on OXPHOS [26] compared with MDA-MB-231, displayed a decrease in OXPHOS upon Disarib exposure. Aiming to disrupt both metabolic pathways, we investigated the combinatorial effect of Disarib with metformin, a well-known complex I inhibitor, on cell proliferation. We assessed the effect in both cell lines as follows: MDA-MB-231 to evaluate the potential for enhanced efficacy and MDA-MB-468 to determine if the combination could further reduce proliferation given the Disarib-mediated decrease in OXPHOS already observed in these cells. MDA-MB-231 and MDA-MB-468 cells were treated with Disarib, 2µM (below IC50) and 4µM (close to IC50), in combination with Metformin 2.5mM (below IC50) and 5mM (close to IC50). The combination showed a significant reduction in the cell viability for MDA-MB-231 and MDA-MB-468 compared to the drug alone. Maximum cell death was observed in 4µM Disarib combined with 5mM Metformin in both the TNBC cell lines (Supplementary Figure S3b.i–ii, c.i–ii). A Chou Talalay test was performed using Compusyn software, (version 5.20.2010) to obtain the drug combination index (CI) values. From the log CI plot, it was evident that there was a synergism between Disarib and Metformin (Supplementary Figure S3d.i–ii).

### 2.7. Disarib Reduced the Expression of Fission Genes in MDA-MB-231 and MDA-MB-468 Cells and Xenografts

**Figure 7:**
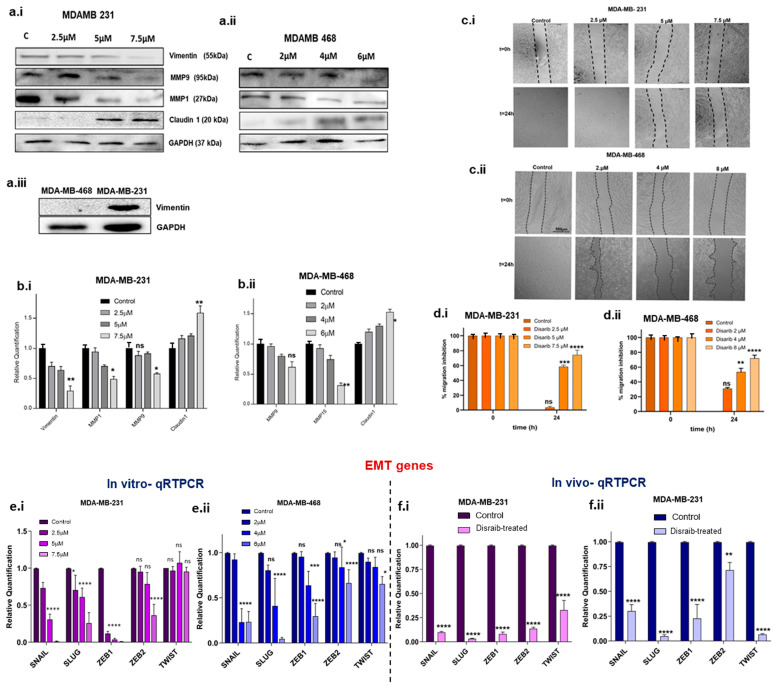
Protein expression and migration assay. (**a.i**) Western blotting image of EMT markers in Disarib-treated MDAMD231. (**a.ii**) Western blotting image of EMT markers in Disarib-treated MDA-MB-468. (**a.iii**) Western blot image of vimentin in control MDA-MB-231 and MDA-MB-468. Quantification of Western blot analysis of (**b.i**) MDA-MB-231 and (**b.ii**) MDA-MB-468. (**c.i**) Representative image of the scratch wound assay for MDA-MB-231. (**c.ii**) qRTPCR of EMT transcription factors from Disarib-treated MDA-MB-468. (**e.i**) qRTPCR of EMT transcription factors from Disarib-treated MDA-MB-231. (**e.ii**) qRTPCR of EMT transcription factors from Disarib-treated MDA-MB-468. (**f.i**) qRTPCR of EMT transcription factors from Disarib-treated MDA-MB-231 xenograft. (**f.ii**) qRTPCR of EMT transcription factors from the Disarib-treated MDA-MB-468 xenograft. The dark purple and blue bars represent control samples, and the light purple and blue bars represent Disarib-treated samples. where ns: non-significant, *p* < 0.05 = *, *p* < 0.01 = **, *p* < 0.001 = ***, *p* < 0.0001 = ****.

Mitochondrial dynamics are interconnected to energy metabolism. A balance between fission and fusion events controls the shape of the mitochondria, which is essential for maintaining the energy demands of cancer cells [28]. We checked the expression of fission and fusion genes in the Disarib- and ABT-199-treated MDA-MB-231 cell line. Log2fold change analysis revealed the downregulation of essential fission genes Mid49, FIS1, DRP1, and MFF1. Fusion genes OPA1 and MFN1 and mitophagy genes PINK1 and PARKIN showed upregulation (Supplementary Figure S3e). To further validate the results, qRTPCR was performed for a panel of mitochondrial dynamics-related genes (Table 1) in Disarib-treated MDA-MB-231, MDA-MB-468 cells, and xenograft tumours. The results suggest that the Mid49, FIS1, DRP1, and MFF1 genes participating in the fission process were downregulated significantly in 7.5µM Disarib-treated MDA-MB-231 (Figure 7a.i). Fusion genes OPA1 and MFN1 and mitophagy genes PINK1 and PARKIN showed upregulation. In the case of Disarib-treated MDA-MB-468 (Figure 6a.ii), DRP1, Mid49, and MFF1 showed downregulation; however, FIS1 was upregulated. MDA-MB-231 showed a pronounced effect on fission genes compared with MDA-MB-468 (Figure 6d.i–ii). OPA1, MFN1, PINK1, and PARKIN1 were also upregu-lated in MDA-MB-468. A qRTPCR was also performed on RNA extracted from control tumour samples and Disarib-treated animals from MDA-MB-231 and MDA-MB-468 cell line-derived xenograft (CDX) models. The results were comparable to the cell lines (Figure 6e.i–ii). However, MDA-MB-231 xenografts had a pronounced effect on fission genes compared with MDA-MB-468. A difference in fusion and fission of mitochondria was observed in the TNBC cell lines, indicating a differential impact of Disarib treatment.

### 2.8. Disarib Downregulated the Expression of Epithelial-to-Mesenchymal Transition Markers and Inhibited the Migration of MDA-MB-231 and MDA-MB-468 Cells

Epithelial-to-mesenchymal transition (EMT) has been linked to mitochondrial dysfunction via metabolic reprogramming. Changes in mitochondrial energy metabolism and dynamics upon Disarib treatment led us to investigate its effect on cell migration. EMT transcription factors are major players in the EMT process and are upregulated in breast cancer. Log2fold changes for EMT-related genes were extracted from Disarib- and ABT-199-treated MDA-MB-231 RNAseq data, and a bar graph was plotted (Supplementary Figure S4a). Further, validation of the results from RNAseq was performed. A qRTPCR was performed to check the levels of EMT transcription factors in the Disarib-treated MDA-MB-231 and MDA-MB-468. Disarib and ABT-199 downregulated all the significant EMT markers and transcription factors observed in RNAseq. Disarib-induced alterations were pronounced compared with ABT-199. In the qRTPCR results of Disarib-treated MDA-MB-231, SNAIL, SLUG, ZEB1, and ZEB 2ZEB2 were significantly decreased compared with the control; however, TWIST1 did not show any change in its expression (Figure 7c,7e.i). In MDA-MB-468, SNAIL, SLUG, ZEB 1, ZEB 2, and TWIST showed a significant decrease in expression (Figure 7c,7e.ii); among the EMT genes, SNAIL and SLUG displayed a maximum reduction in the levels in Disarib-treated samples compared with the control. We also validated the SNAIL, SLUG, ZEB1, ZEB2, and TWIST levels in CDX models. Tumours from the control and treatment groups were collected in trizol, RNA was isolated, complementary DNA (cDNA) was prepared, and a qRTPCR was performed. The results show that in both MDA-MB-231 and MDA-MB-468 xenografts, EMT transcription factors were downregulated significantly in Disarib-treated samples compared with the control. In MDA-MB-231 xenografts (Figure 7d,7f.i), SNAIL, SLUG, ZEB1, and ZEB2 were the most downregulated whereas, in MDA-MB-468 xenografts (Figure 7d,7f.ii), SLUG and TWIST were more significantly downregulated than the others. These results correlated with the Disarib-treated cell line qRTPCR data.

The alterations in EMT markers observed at the transcript level were tested at the protein level using Western blotting upon Disarib treatment after 48 h in MDA-MB-231 and MDA-MB-468. Mesenchymal markers including vimentin, extracellular matrix-degrading metalloproteinase levels, and claudin were examined (Figure 7a.i–ii). Vimentin is a mesenchymal marker expressed abundantly in basal MDA-MB-231 and is not expressed in basal MDA-MB-468 (Figure 7a.iii). Upon Disarib treatment, vimentin protein levels showed a significant concentration-dependent reduction in MDA-MB-231. Claudins are essential components of tight junctions. In TNBC, claudin expression is primarily low. Low claudin TNBC cells are more aggressive. We checked the protein levels of claudin upon Disarib treatment in MDA-MB-231 and MDA-MB-468, and the results showed that claudin expression increased in a concentration-dependent manner in both the TNBC cell lines. At the highest concentration of Disarib (7.5µM for MDA-MB-231 and 6µM for MDA-MB-468), an increase in the expression of claudin was observed for both MDA-MB-231 and MDA-MB-468 (Figure 7a.i-ii). MMP1 and MMP9 are matrix metalloproteases that degrade the extracellular matrix and facilitate migration. They are overexpressed in TNBC and are correlated with poor prognosis. Upon Disarib treatment, MMP1 and 9 were significantly downregulated in a concentration-dependent manner in the MDA-MB-231 and MDA-MB-468 cell lines (Figure 7bi–ii). CD44 is a cell surface adhesion receptor marker for cancer stemness and metastatic potential [7]. An immunofluorescence assay was performed to examine the levels of the CD44 marker upon Disarib treatment in MDA-MB-231 and MDA-MB-468 cells (Supplementary Figure S4b). MDA-MB-231 control cells had higher expression of CD44 compared with MDA-MB-468. Disarib treatment showed a reduction in CD44 staining in both cell lines. 

EMT markers and transcription factor expression were downregulated upon Disarib treatment. Therefore, we further investigated the effect of Disarib on the migration of MDA-MB-231 and MDA-MB-468. Firstly, we performed a scratch wound assay. MDA-MB-231 and MDA-MB-468 were seeded in a 12-well plate and grown for 24 h; using a small tip, a scratch was made, and then media with reduced serum were added and monitored for migration. In 12 h, MDA-MB-231 cells had migrated and covered the scratch area in the control cells, whereas, at 5µM and 7.5 µM, wound closure did not occur (Figure 7c.i,d.i). In the case of MDA-MB-468, the control cells closed the wound in 24 h. The Disarib-treated cells (4 µM and 6 µM) did not migrate. The scratch remained intact at 24 h, suggesting that migration was inhibited in these two cell lines upon Disarib treatment (Figure 7c.ii,d.ii).

## 3. Discussion

This study investigated the cytotoxicity of Disarib in TNBC cell lines. The observed IC_50_ values ranged from 4.3 to 7.7 µM, with MDA-MB-468 exhibiting the highest sensitivity and MDA-MB-453 showing the lowest. This finding correlated with BCL2 protein levels, which were relatively higher in MDA-MB-231 and MDA-MB-468 than in MDA-MB-453 [29]. MDA-MB-231 displayed higher BCL-xL levels than MDA-MB-468, potentially explaining the observed IC_50_ difference despite similar BCL2 levels. The expression and cellular localisation of proapoptotic proteins, BAX and BAK, also influence cell death susceptibility. While both cell lines expressed BAX and BAK, their ratios differed slightly. MDA-MB-231 exhibited higher BAK levels, while MDA-MB-468 had higher BAX protein. Notably, BAK primarily localises to the mitochondria, the site of apoptosis initiation, whereas BAX resides in the cytosol and requires translocation for action [30,31]. This suggests that Bak in MDA-MB-231 might trigger cell death at lower Disarib concentrations than cytosolic BAX in MDA-MB-468, potentially contributing to the subtle IC_50_ variation.

The colony-forming assay tests the ability of single cells to form colonies, indicating stemness [24]. This study revealed that Disarib treatment exhibited a more significant reduction in colony formation for MDA-MB-468 cells than for MDA-MB-231 cells. This finding correlates with the differences in antiapoptotic proteins BCL2 and BCL-xL expression levels. The higher level of Bcl-xL in MDA-MB-231 cells might contribute to its stem cell-like properties. Previous research suggests Bcl-xL expression in mammary cells can promote stemness through RAS signalling [32]. Additionally, nuclear localisation of BCL2 and BCL-xL has been reported in breast cancer, indicating their potential role in regulating stemness [33]. The overexpression of antiapoptotic BCL2 family proteins is associated with epithelial-to-mesenchymal transition (EMT), chemoresistance, and stemness in cancer cells. These proteins decrease apoptosis through the modulation of mitochondrial outer membrane permeability (MOMP) [34]. High levels of BCL2 proteins prevent the release of cytochrome c, a critical step in apoptosis, by inhibiting the formation of the apoptosome complex, which activates caspases 3 and 9. Our data demonstrate that Disarib treatment in TNBC cells triggers cell death by altering MOMP and inducing caspase activation. Proapoptotic members of the *BCL2* family, such as BAX, are essential for mitochondria-mediated apoptosis [35]. BAX activation is regulated by the tumour suppressor protein TP53, which is involved in MOMP [36]. However, MDA-MB-231 and MDA-MB-468 cells harbour mutations in the p53 gene [37]. Disarib offers a potential therapeutic advantage by targeting the BCL2/BAK interaction at the BH1 domain, a mechanism independent of TP53 regulation [19]. However, recent reports suggest the emergence of chemoresistance to ABT-199, potentially because of altered BAX activity in TP53-mutant backgrounds [38]. To counteract the effects of BH3 domain interactors, the BH1 domain interactors of BCL2, not regulated by TP53, can be targeted. Disarib targets the BCL2/BAK interaction at the BH1 domain to alter MOMP and cell death [21]. Our findings demonstrate that Disarib exhibits greater efficacy in TP53-mutant TNBC cells. Additional experiments on the TP53 mutant background are required to validate the impact of Disarib.

One of the markers of loss in mitochondrial outer membrane permeability is the generation of ROS [39]. ROS-inducing agents increase the ROS level, disrupt redox homeostasis, and trigger cancer cell death [40]. Consistent with these observations, Disarib treatment elevated ROS levels in TNBC cells. ROS-induced oxidative stress is a potent inducer of cell death through various pathways, including apoptosis, necroptosis, and autophagy [41]. Our findings demonstrate that Disarib triggers apoptosis in TNBC cells via the intrinsic pathway. This is evidenced by the upregulation of proapoptotic proteins Bak, Apaf-1, and cytochrome c, alongside the downregulation of the anti-apoptotic protein BCL2 and inactive forms of caspase 3 and caspase 9. This proapoptotic/anti-apoptotic protein balance shift tilts the cell towards programmed cell death. Interestingly, Disarib-mediated cell death did not precede cell cycle arrest. This observation aligns with the mutated TP53 status of the TNBC cell lines used, as TP53 dysfunction is often associated with bypassing cell cycle checkpoints during apoptosis. 

Disarib showed better activity on the TP53-mutated metastatic TNBC cell lines and undifferentiated cells with cancer stem-like properties than the TP53 wildtype cell line MCF7 (Supplementary Figure S1b.i–ii). RNAseq analysis of the Disarib-treated samples revealed upregulation of ETC complex I. Metformin targets the ETC I complex and has shown promising results in treating cancers through combination therapy. In breast cancer, the results have not been promising. However, Metformin and other chemotherapeutic drugs in some cancer subtypes showed better outcomes [42]. Disarib displayed a synergistic effect on cancer cell death when combined with Metformin. This warrants further investigation into the potential therapeutic benefit of combining Disarib with Metformin for TNBC treatment. The ongoing clinical trials exploring Metformin’s role in combination therapy for various cancers provide further impetus for such research [42]. Disarib’s preclinical findings are encouraging and pave the way for future studies. It is noteworthy that ABT-199 (Venetoclax), another BCL2 inhibitor targeting the BH3 domain and disrupting the BCL2/BAX interaction, has demonstrated synergy with other drugs in Acute Myeloid Leukaemia (AML) [43]. This highlights the potential of combining Disarib with other therapeutic agents for a more robust treatment strategy in TNBC.

One major factor that induces EMT and drug resistance is the metabolic reprogramming of cancer cells [11]. This study acknowledges the established link between EMT and metabolic reprogramming in cancer cells. Cancer cells utilise glycolysis even in aerobic conditions to rapidly produce energy for cell proliferation and metastasis [10]. TNBC cells exhibit a unique metabolic plasticity, utilising glycolysis and oxidative phosphorylation (OXPHOS) for energy production [9]. This work highlights the heterogeneity in TNBC, with BCL2-positive MDA-MB-231 cells leaning towards high glycolysis and lower OXPHOS, while MDA-MB-468 cells exhibit the opposite phenotype [26]. Among luminal cells, MCF7 is known to have a high glycolytic rate compared with T47D, which has negligible BCL2 [44]. However, in T47D, glycolysis is the primary energy metabolic pathway. Overexpression of glycolytic enzymes HK1, PGK1, and PGAM1 is observed in TNBC cells [45]. Disarib-treated MDA-MB-231 cells showed inhibition of glycolytic enzymes, which was not observed in BCL2-negative T47D cells, indicating BCL2-specific regulation of gene expression. However, OXPHOS did not show a significant reduction in RNAseq data. The ECAR assay showed that Disarib reduced glycolytic activity at 24 and 48 h in MDA-MB-231 and MDA-MB-468 cells. Disarib-treated MDA-MB-468 showed better inhibition of OXPHOS. High concentrations of ABT-737 reduce mitochondrial ATP synthesis in malignant cells [46]. Venetoclax, an FDA-approved BCL2 inhibitor, has been shown to inhibit mitochondrial respiration in leukemic stem cells [47]. Venetoclax, in combination with azacytidine, blocked the TCA cycle and OXPHOS in leukemic stem cells [48]. In venetoclax-resistant leukemic cells, glycolysis is upregulated, indicating that metabolic reprogramming might account for drug resistance [49]. Chemoresistance due to metabolic reprogramming can be circumvented by combination therapy of BCL2 and metabolic inhibitors. Biguanides sensitise ABT-737-induced cell death by inhibiting OXPHOS [50]. Venetoclax and Metformin synergise in acute myeloid leukaemia by downregulating MCL1 [43]. ABT-199 sensitises cells to the AMPK inhibitor acadesine [51]. 

Mitochondrial dynamics are interconnected with energy metabolism. A balance between fission and fusion events controls the shape of mitochondria, which is vital for maintaining the energy demands of the cancer cell [28]. Well-formed mitochondria and their complexes are seen in cells with high energy demand. In contrast, elongated mitochondria with diffused complexes are seen in cells with low energy demand [52]. Patients with TNBC show pronounced mitochondrial fission and are often associated with poor survival. A major player in fission, *DRP1* is highly expressed in TNBC, thereby causing an increase in the metastatic potential of cells. *DRP1* is overexpressed during the hypoxic condition in MDA-MB-231 cells. DRP-1 is essential for BH3 mimetics-induced apoptosis and mitochondrial fragmentation [53]. Critical genes involved in fusion and mitophagy were upregulated upon Disarib treatment. TNBC showed lower levels of MFN1, a fusion protein [54]. Low levels of MFN2 are associated with nodal metastasis in TNBC [55]. Activation of mitophagy occurs under stress conditions to conserve nutrients and reduce mitochondrial mass [40]. RNA sequencing and single-cell imaging showed that overexpression of BCL2 or BCL-xL elevates mitochondrial dynamics by moderating the gene expression in this pathway [56]. ABT-737 induces apoptosis and mitophagy by modifying mitochondrial fission in ovarian cancer cells [57]. ABT-737 increases sensitivity to cisplatin by modulating mitochondrial dynamics in cholangiocarcinoma [58].

EMT is a process with a change in the epithelial-to-mesenchymal phenotype, helping cells metastasise. The cells gain mesenchymal markers like vimentin, and a shift in the gene expression pattern is observed where N-cadherin expression replaces E-cadherin expression moderated by transcription factors ZEB and SNAIL [59]. Further, mesenchymal cells gain stem cell-like properties, express CD44, and have low CD24 levels [60]. Additionally, BCL2 overexpression has been shown to activate the EMT programme [61]. In our study, Disarib, a BCL2 inhibitor, reduced the expression of EMT transcription factors SNAIL, ZEB, and SLUG in MDA-MB-231 and MDA-MB-468 cells. In patients with TNBC, ZEB1 overexpression is related to poor prognosis [6], and SNAIL and Twist are known to induce stemness in TNBC. Disarib also downregulated matrix metalloproteases MMP1 and MMP9 and the mesenchymal marker vimentin. Increased vimentin expression leads to migration in TNBC cell lines [62]. MMP1 and MMP9 are overexpressed in TNBC cells [63]. The drug resistance observed in doxorubicin treatment is due to the activation of EMT via the PI3/AKT and SMAD pathway in TNBC cells [64]. Several drugs have been known to alter the EMT pathway in cancer cells [65]. Cisplatin is known to block EMT in breast cancer cells by reducing the expression of EMT transcription factors [66]. Tamoxifen is known to reverse EMT characteristics in mesenchymal cells [67]. Metabolic inhibitors Metformin and 2DG have been known to block migration by bringing down the EMT in TNBC cells [64]. Disulfiram, an ALDH1A inhibitor, has been shown to block the EMT pathway in hepatocellular carcinoma [68]. Natural products like curcumin and its derivatives have inhibited migration by regulating EMT processes in breast cancer cells [69]. Vimentin is abundant in Basal B cell line MDA-MB-231, whereas it has lower expression in MDA-MB-468. The impact of BCL2 inhibitors on EMT has not been evaluated. This study is the first study showing a connection between BCL2, mitochondrial function, and EMT.

In conclusion, our study demonstrates Disarib’s multifaceted approach against TNBC. Disarib disrupts the interaction between BCL2 and proapoptotic BAK protein, triggering cell death via the intrinsic apoptotic pathway in TNBC cells. Furthermore, Disarib disrupts mitochondrial function by downregulating key glycolytic genes and mitochondrial fission genes, potentially hindering tumour growth and metastasis. Additionally, Disarib suppresses epithelial-to-mesenchymal transition, a process linked to metastasis. These findings highlight Disarib’s potential as a promising therapeutic strategy for TNBC by targeting cancer cell survival, metabolism, and metastatic potential.

## 4. Materials and Methods

### 4.1. Chemicals and Reagents

All the chemicals used in the experiments were purchased from Biorad (Hercules, CA, USA), Thermo Fisher Scientific (Waltham, MA, USA), MP Biomedicals (Irvine, California, USA), Life Technologies (Carlsbad, CA, USA), KAPA (Basel, Switzerland), Sigma Chemical Co. (St. Louis and Burlington, MA, USA), and BioLegend (San Diego, CA, USA). Primers were ordered from Bioserve Biotechnologies India Pvt Ltd. NGS reagents were purchased from Illumina (San Diego, CA, USA) and New England Biolabs (Ipswich, MA, USA). Cell lines were purchased from the National Centre for Cell Sciences, India (NCCS, Pune, India). Disarib was synthesised in SCR lab, IISc, Bengaluru.

### 4.2. Cell Culture

MDA-MB-231, MDA-MB-468, and MDA-MB-453 were grown in DMEM and T47D and CEM in RPMI 1640 medium supplemented with 10% foetal bovine serum (FBS), 100 µg/mL Penicillin, and 100 µg streptomycin/mL and incubated at 37 °C in a humidified atmosphere containing 5% CO_2_.

#### 4.2.1. MTT Assay

Cytotoxicity exerted by Disarib on TNBC cell lines was assessed by the 3-(4,5-dimethylthiazol-2-yl)-2,5-diphenyltetrazolium bromide assay (MTT assay). Briefly, a 96-well plate with a cell density of 5000 cells/well was treated with an increasing concentration of Disarib in triplicates and incubated for 24 and 48 h. Post incubation, 10 µL of MTT (5 mg/mL) was added to each well and incubated until a purple-coloured formazan was formed. The absorbance of solubilised formazan crystals was read at 570 nm. Dimethyl sulphoxide (DMSO) was used as vehicle control. The IC_50_ of the compounds was obtained from the 48 h treatment using Graphpad Prism software version 7.0, (accessed on 1 April 2022).

#### 4.2.2. Lactate Dehydrogenase (LDH) Assay

This assay measures the stable Lactate dehydrogenase in the cytosol. To perform this assay, 5000 cells were seeded in a 96-well plate in triplicates and were treated with increasing concentrations of Disarib. After 24 and 48 h of treatment, the cells were lysed using 0.5% Triton-X-100. Then, 5 µL of the lysate from each well was mixed with LDH reagents. The orange-red-coloured formazan product obtained was measured at 490 nm. Dimethyl sulfoxide (DMSO)-treated cells acted as vehicle control.

### 4.3. Colony-Forming Assay

MDA-MB-231, MDA-MB-468, and MDA-MB-453 cells were seeded in wells at a density ranging from 500 to 1000 cells per well. After overnight incubation, the cells were exposed to varying concentrations of Disarib tailored to each cell line as follows: MDA-MB-231: 2.5, 5, 7.5, and 10 µM, MDA-MB-468: 2, 4, 6, and 8 µM and MDA-MB-453: 8, 10, 12, and 14 µM. Following a 10-day incubation period, the culture medium was removed, and the wells were washed with phosphate-buffered saline (PBS) at 1X concentration. Crystal violet staining (0.2%) was used to visualise the colonies. Images were captured, and the colony area was quantified using ImageJ software (version 1.52a) with the Colony Area plugin21 (software version 1.52a, https://imagej.nih.gov/ij/ (accessed on 5 March 2024).

#### 4.3.1. AnnexinV-FITC and PI Double Staining to Examine Apoptosis

To assess apoptosis or necrosis, Annexin V-FITC/PI double staining was performed in the TNBC cell lines MDA-MB-231, MDA-MB-468, and MDA-MB-453 treated with Disarib [70,71]. MDA-MB-231 was treated with 5, 7.5, and 10 µM of Disarib, MDA-MB-468 was treated with 4, 6, and 8 µM of Disarib, and MDA-MB-453 was treated with 10, 12, and 14 µM of Disarib. DMSO was used as the vehicle control. The treatment was performed for 48 h and then stained with Annexin V-FITC and PI for 20 min and run in a flow cytometer (BC gallios) using Gallios, Beckman Coulter software, (Miami, FL, USA, version 1.2, https://www.mybeckman.in/) at an excitation with 488 nm laser and emission at 530 nm for FITC and 617 for PI. A minimum of 10,000 events were acquired per sample. Data were analysed using Flowing Software (version 2). The experiments were repeated at least three independent times, and the data are presented along with error bars.

#### 4.3.2. JC1 Assay to Investigate Mitochondrial Membrane Potential

The effect of Disarib on mitochondrial membrane potential was monitored in a flow cytometer using JC1 dye (5,5′,6,6′-tetrachloro-1,1′,3,3′-tetraethylbenzimidazolylcarbocyanine iodide). The shift from red to green fluorescence depicts changes in mitochondrial membrane potential [72]. Briefly, MDA-MB-231, MDA-MB-468, and MDA-MB-453 were grown in 6-well plates with a cell density of 75,000 cells/mL and treated with Disarib after 24 h. MDA-MB-231 was treated with 5, 7.5, and 10 µM of Disarib, MDA-MB-468 was treated with 4, 6, and 8 µM of Disarib, and MDA-MB-453 was treated with 10, 12, and 14 µM of Disarib. After 48 h of treatment, the cells were harvested and resuspended in the staining buffer containing JC1 for 20 min at 37 °C, 5% CO_2_ in the dark. The cells were then subjected to flow cytometry, and 10,000 events were acquired.

#### 4.3.3. Cell Cycle Analysis

DNA content was analysed by staining with PI to understand the cell cycle phases. MDA-MB-231, MDA-MB-468, and MDA-MB-453 were grown in 6-well plates with a cell density of 75,000 cells/mL and treated with Disarib after 24 h. MDA-MB-231 was treated with 5, 7.5, and 10 µM of Disarib, MDA-MB-468 was treated with 4, 6, and 8 µM of Disarib, and MDA-MB-453 was treated with 10, 12, and 14 µM of Disarib. After 48 h of treatment, the cells were harvested, resuspended in citrate buffer (0.1% trisodium citrate, 0.03% NP-40, 100 ug/mL RNase A), and incubated at 37 °C for 15 min. PI (4 µg/mL) was added, and the events were acquired by the FL3 channel of the flow cytometer (Gallios, Beckman Coulter, Miami, FL, USA and the in- built Gallios software (version 1.2, https://www.mybeckman.in/)).

### 4.4. Reactive Oxygen Species (ROS Analysis)

#### 4.4.1. Spectrophotometric ROS Assay

MDA-MB-231, MDA-MB-468, and MDA-MB-453 cells were seeded at a density of 20,000 cells/well on an opaque 96-well plate. An increasing concentration of Disarib was added. Post 24 h, the cells were washed in 1X PBS and incubated with H2DCFDA (10 µM). ROS scavenger N-acetylcysteine (NAC) (20 mM) was added in 1X PBS for 1 h. Post incubation, the fluorescence was measured at ex 485/em 535 using a fluorescent microplate reader. In this experiment, 200 µM H_2_O_2_ was used as a positive control.

#### 4.4.2. Flow Cytometric Reactive Oxygen Species Assay

Reactive oxygen species (ROS) levels were also assessed using a flow cytometer. The cells were stained with H2DCFDA (2’,7’-dichlorodihydrofluorescein diacetate [73,74]. MDA-MB-231, MDA-MB-468, and MDA-MB-453 were grown in 6-well plates with a cell density of 75,000 cells/mL and treated with Disarib after 24 h. MDA-MB-231 was treated with 5 µM, MDA-MB-468 was treated with 4 µM, and MDA-MB-453 was treated with 7.7 µM of Disarib for 2 h, 30 min, and 4 h, respectively. The cells were harvested and washed with PBS. H2DCFDA staining was performed for 30 min and analysed by flow cytometry. H_2_O_2_-treated cells were used as a positive control. ROS measurement was analysed based on the degree of shift in the histogram by considering median fluorescence intensity as a parameter.

### 4.5. Western Blotting Analysis

To check the effect of Disarib on protein expression in MDA-MB-231 and MDA-MB-468, cells were grown in 6-well plates with a cell density of 75,000 cells/mL and treated with Disarib after 24 h. MDA-MB-231 was treated with 5, 7.5, and 10 µM of Disarib, and MDA-MB-468 was treated with 4, 6, and 8 µM of Disarib. The cells were harvested after 48 h of treatment and washed with 1X PBS. Protein was extracted using RIPA buffer. Around 40 µg of protein was loaded on 8–10% SDS PAGE and transferred to a PVDF membrane (Biorad, Hercules, CA, USA). The membrane was blocked using 5% skimmed milk powder (1 h, RT) and probed with appropriate primary antibodies (Santa Cruz, CA, USA and CST, Danvers, MA, USA) for different proteins (BCL2, Apaf1, Bak, Procaspase 3, cytochrome c, Tubulin, vimentin, Mmp1, Mmp9, claudin 1). The blots were washed in PBST (1X PBS and 0.1% Tween 20) and incubated with HRP-conjugated secondary antibodies (Santa Cruz; 1:10,000) at room temperature for 2 h. The blots were rinsed in PBST, developed using a chemiluminescent solution (Biorad, Clarity^TM^ Western ECL substrate. Hercules, CA, USA), and scanned by a gel documentation system (Biorad, Hercules, CA, USA). Protein band image quantification was performed using GelQuant.Net (version 1.7.8), http://biochemlabsolutions.com/GelQuantNET.html (accessed on 10 January 2022).

### 4.6. Scratch Wound Assay

MDA-MB-231 and MDA-MB-468 cells were seeded at 80,000 cells/well in a 24-well plate. After 24 h, wounds were created using a pipette tip; the scraped cells were removed with PBS wash. The cells were then incubated in 2% serum media with/without Disarib. MDA-MB-231 was treated with 5, 7.5, and 10 µM of Disarib, and MDA-MB-468 was treated with 4, 6, and 8 µM of Disarib. Images were obtained at different time points; the wound area was quantified manually using ImageJ software (version 1.52a), https://imagej.nih.gov/ij/ (accessed on 11 January 2022) and the percentage wound closure area was plotted as a bar graph.

### 4.7. Immunofluorescence

MDA-MB-231 and MDA-MB-468 cells were seeded at 75,000 cells/well in a 6-well plate with coverslips. After 24 h, MDA-MB-231 was treated with 5 µM, and MDA-MB-468 was treated with 4 µM. After 48 h of treatment, cells were washed and fixed in 2% paraformaldehyde and incubated for 15 min. The cells were then permeabilised with 0.1% Tween20 in PBS and blocked in 5% BSA for an hour. CD44-FITC (Biolegend, San Diego, CA, USA) was used at a concentration of 1:200 and incubated for 1 h at RT. After washing with 1X PBS, the coverslip was mounted using slow-fade antifade reagent containing DAPI (Thermo Fisher Scientific, Waltham, MA, USA), and images were captured using a fluorescent microscope.

#### 4.7.1. Oxygen Consumption Rate Assay

Oxidative phosphorylation occurs in mitochondria in the presence of oxygen to generate ATP. The oxygen consumption rate (OCR) measurement indicates whether a cell depends on OXPHOS. The OCR assay kit from Cayman was used to check whether Disarib affects energy metabolism. MDA-MB-231 and MDA-MB-468 cells were seeded at a density of 100,000 cells/mL in a 6-well plate to perform the assay. After 24 h, the IC_50_ concentration of Disarib was added to both the cell lines and DMSO was added to the control well. The cells were incubated for 24 and 48 h. Post incubation, 80,000 cells were counted and seeded in an opaque 96-well plate from control and treatment wells for both the cell lines. Antimycin-treated cells were used as a control. A phosphorescent probe (10 µL) was added to each well, and the fluorescence was measured for 4 h in molecular devices spectraMax instrument with the wavelength setting of 380 nm for excitation and 650 nm emission. The cells from each well were lysed, and protein was estimated using the Bradford assay, which was used for normalisation. Percent normalised fluorescence intensity was plotted as a bar graph.

#### 4.7.2. Extracellular Acidification Rate Assay

Cells utilising glycolysis have an acidic medium because of lactic acid production. Therefore, a measure of acidification indicates that the cells depend on glycolysis. An extracellular acidification rate (ECAR) assay was performed using a kit from Abcam [75]. MDA-MB-231 and MDA-MB-468 cells were seeded at a density of 100,000 cells/mL in a 6-well plate to perform the assay. After 24 h, the IC_50_ concentration of Disarib was added to both the cell lines and DMSO was added to the control well. The cells were incubated for 24 and 48 h. Post incubation, materials and reagents used for the assay were purged to avoid any acidification due to CO_2_. Then, 80,000 cells were counted and seeded in an opaque 96-well plate from control and treatment wells for both cell lines. A glycolysis assay reagent (10 µL) was added to each well, and the fluorescence was measured for 4h in molecular devices spectraMax instrument with the wavelength setting of 380 nm for excitation and 615 nm emission. The cells from each well were lysed, and protein was estimated using the Bradford assay, which was used for normalisation. Percent normalised fluorescence intensity was plotted as a bar graph.

#### 4.7.3. Human Cell Line-Derived Xenograft Models

A total of 40 Swiss albino nude mice were used for this study. Among these, 20 were injected subcutaneously with MDA-MB-231 cells (5 × 10^6^ cells in 1% Matrigel/animal). The remaining 20 animals were injected subcutaneously with MDA-MB-468 cells (5 × 10^6^ cells in 1% Matrigel/animal). The animals were divided into MDA-MB-231 and MDA-MB-468. Group I served as tumour control and received no treatment. Group II (MDA-MB-231) and Group III (MDA-MB-468) received 12 doses of 50 mg/kg Disarib administered orally daily. The tumour growth was monitored by measuring the diameter of the tumour using vernier callipers every alternate day. The tumour volume was calculated using the formula V = 0.5 × a × b2, where “a” and “b” indicate major and minor diameters, respectively. The ethics approval number for this study is IAEC/IBAB/08/12/2019.

#### 4.7.4. Library Preparation and Sequencing

MDA-MB-231 cells were cultured in DMEM, and 75,000 cells/mL were seeded in a 6-well plate and treated with the IC_50_ concentration of Disarib and ABT-199 48 h. Then, 75,000 cells/mL of T47D cells were seeded in a 6-well plate and treated with Disarib for 48 h. The samples were then collected after incubation, and trizol was added. RNA was isolated using the standard Trizol method. RNA was quantified using Qubit, and the quality was checked on a tape station. mRNA libraries were prepared using Illumina TruSeq RNA Library Prep Kit v2 (San Diego, CA, USA). Briefly, mRNA was isolated using oligo-dT beads (Illumina, San Diego, CA, USA), followed by fragmentation. Fragmented RNA was then converted to cDNA, and adaptor ligation was performed. Size selection was performed on adaptor-ligated libraries using ampure beads (Beckman Coulter, Brea, CA, USA). The libraries were then amplified and checked on a tape station to determine the library size [76,77]. A pool of libraries was prepared and loaded onto the flow cell.

#### 4.7.5. Processing, Alignment of Fastq Files, and Differential Analysis

The samples were sequenced in-house using Illumina Hiseq2500 to acquire 100 bp paired-end reads. The samples had reads greater than 10 million. The quality of the reads was checked using the FastQC tool. The reads were then aligned to the reference hg38 (downloaded from The University of California, Santa Cruz (UCSC) genome browser) for human samples and mm10 reference for mouse samples using bowtie2 with default parameters. A SAM (sequence alignment map) format file was obtained as an output of bowtie2. A binary alignment map (BAM) file was obtained using Samtools [78] from the SAM file. The hg38refseq.bed annotation file was downloaded from UCSC for humans and mm10refseq.bed for mice, and read counts were generated using bed tools [79]. The read counts were quantile-normalised using the R package. Normalised read counts were subjected to differential analysis. In MDA-MB-231 and T47D, differential analysis was performed between the control and Disarib/ABT-199-treated samples. A cutoff of p-value adjusted less than 0.05 and log2 fold change (<−1 and >+1) was applied to obtain a significant DEG list from MDA-MB-231-treated Disarib, ABT-199, and T47D-treated Disarib data. A heatmap was plotted to analyse genes from metabolic pathways using pheatmap, an R package, https://cran.r-project.org/, version R-4.4.0 (accessed on 10 December 2021).

### 4.8. First-Strand cDNA Synthesis and Real-Time PCR

Complementary DNA was synthesised using NEB reagents from the intact RNA using a standard protocol. Real-time PCR was conducted using SYBR^®^ Green chemistry. Primers for BCL2, EMT marker genes, fission, fusion, and mitophagy genes were used with GAPDH primer as an internal control. The initial denaturation was performed at 95°C for 5 minutes, followed by the cycling stage (40 cycles, 95 °C for 20 s, 53 °C for 20 sec, 72°C for 20 s) and melt curve stage. The relative gene expression was calculated by correlating the expression of the housekeeping gene and the expression of the target gene in the control/normal sample. Graphs showing relative quantification for all the samples were plotted using Graphpad Prism software version 7.0 (accessed on 1 April 2022).

### 4.9. Statistical Analysis

Statistical analyses and graphing were performed using GraphPad Prism 7.0 software (GraphPad, San Diego, CA, USA) and R packages https://cran.r-project.org/, (version R-4.4.0), (accessed on 10 December, 2021) [80]. Deseq2 uses the Wald test statistic with a probability to generate a significant gene list. The Benjamini–Hochberg False Discovery Rate (FDR) method was used to choose significant pathways from the Reactome database (https://reactome.org/). For comparative qRTPCR analysis, a two-tailed t-test was applied to calculate the significance. *p*-values less than 0.05 were considered significant, and the bar graphs are represented as mean +SEM. *p* < 0.05 = *, *p* < 0.01 = **, *p* < 0.001 = ***, *p* < 0.0001 = **** were used for representation.

## 5. Conclusions

Disarib demonstrates a promising multifaceted approach for treating TNBC by targeting BCL2 and disrupting cancer cell survival, metabolism, and migration. This study lays the groundwork for further investigating Disarib as a potential therapeutic option for patients with TNBC. The findings indicating Disarib’s impact on metabolism and epithelial-to-mesenchymal transition pathways pave the way for further investigation into combination therapies targeting different aspects of cancer cell survival and metastasis. Beyond TNBC, this research paves the way for exploring BCL2-targeted therapies in other cancers with BCL2 overexpression.

## Figures and Tables

**Table 1:** Primers used in this study.

Gene	Forward (5′-3′)	Reverse (5′-3′)
SNAIL	AATCGGAAGCCTAACTACAGCG	GTCCCAGATGAGCATTGGCA
SLUG	AAGCATTTCAACGCCTCCAAA	AGGATCTCTGGTTGTGGTATGAC
ZEB1	GGGAGGAGCAGTGAAAGAGA	TTTCTTGCCCTTCCTTTCTG
ZEB2	AAGCCAGGGACAGATCAGC	CCACACTCTGTGCATTTGAACT
TWIST1	GTTGACTCTAGCTCGGACCAC	GCCAGTTTGATCCCAGTATTTT
Mid49	CAGAAACGGGGGAAGCGG	CACCAGGAGACGCACATGG
MFN1	GAGGTGCTATCTCGGAGACAC	GCCAATCCCACTAGGGAGAAC
MFN2	CACATGGAGCGTTGTACCAG	TTGAGCACCTCCTTAGCAGAC
OPA1	TGTGAGGTCTGCCAGTCTTTA	TGTCCTTAATTGGGGTCGTTG
DRP1	ACCCGGAGACCTCTCATTCT	TGACAACGTTGGGTGAAAAA
FIS1	GATGACATCCGTAAAGGCATCG	AGAAGACGTAATCCCGCTGTT
MFF	CACCACCTCGTGTACTTACGC	GTCTGCCAACTGCTCGGATTT
PINK1	CCCAAGCAACTAGCCCCTC	GGCAGCACATCAGGGTAGTC
PARKIN	GTGTTTGTCAGGTTCAACTCCA	GAAAATCACACGCAACTGGTC
GAPDH	CCCTTCATTGACCTCAACTACAT	CTGGAGATGGTGATGGGATTT

## Data Availability

Data will be available upon request.

## Supplementary Materials

The following supporting information can be downloaded at: https://www.mdpi.com/article/10.3390/ijms25126485/s1.
