# Supplementary material for: Disarib, a Specific BCL2 Inhibitor, Induces Apoptosis in Triple-Negative Breast Cancer Cells and Impedes Tumour Progression in Xenografts by Altering Mitochondria-Associated Processes
Source: Int J Mol Sci. 2024 Jun 12;25(12):6485. doi: 10.3390/ijms25126485 (PMC11203414; doi:10.3390/ijms25126485)
Supplement: Supplementary file 1 [file ijms-25-06485-s001.zip › Supplementary figures_revised_2.docx]

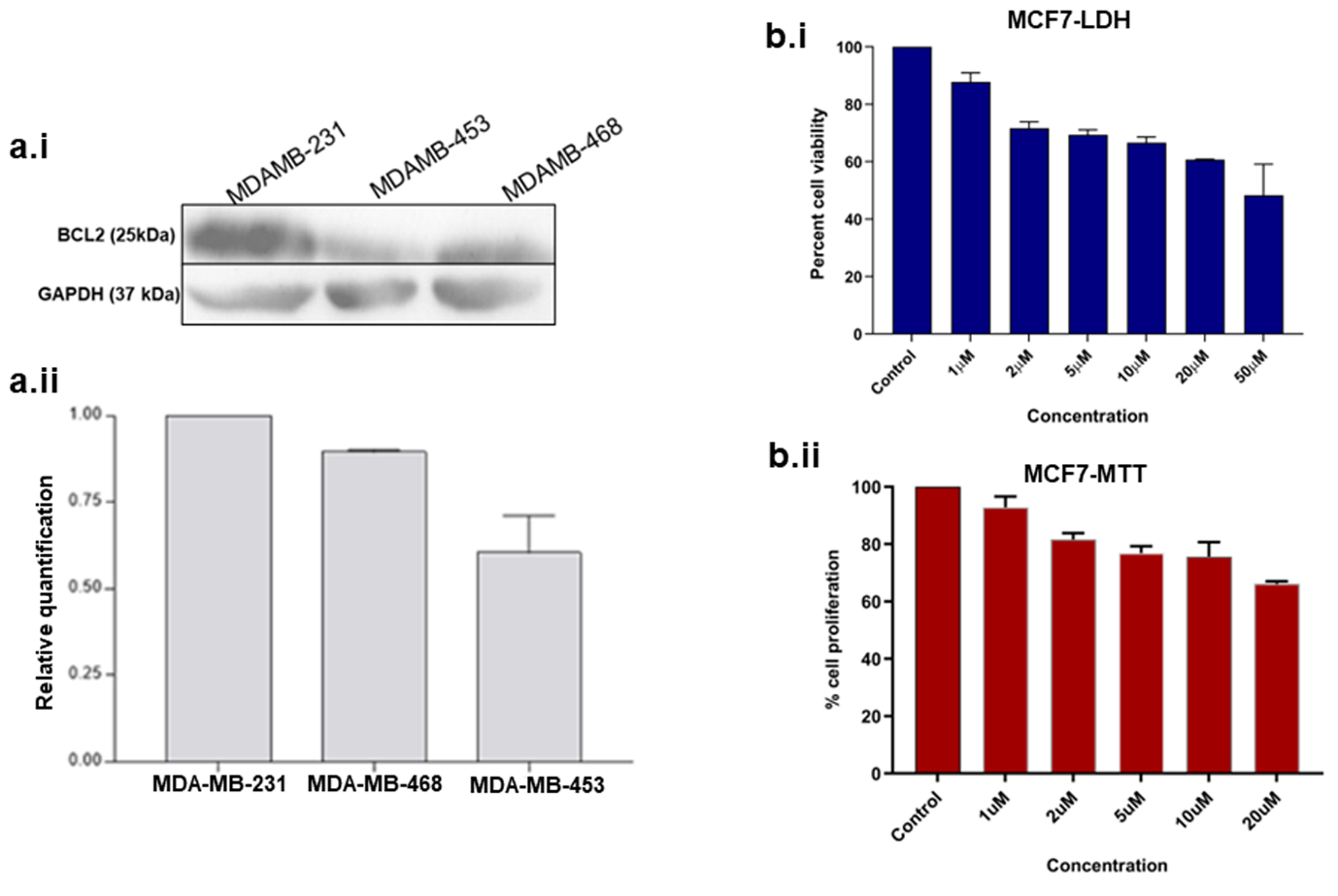
**Supplementary figures**

**Figure S1: a.i** Western blotting to check Bcl2 expression levels in triple negative breast cancer cell lines MDA-MB-231, MDA-MB-468, and MDA-MB-453. **a.ii**. Quantification. bar graphs for the Bcl2 level in MDA-MB231, MDA-MB-468 and MDA-MB-453 cell lines. **b.i** LDH assay graph for MCF7 **b.ii**. MTT assay graph for MCF7. The error bars represent the standard error of the mean (SEM). Statistical significance is indicated by asterisks: *, p < 0.05 = *, p< 0.01 = **, p<0.001 = ***, p < 0.0001 = ****)


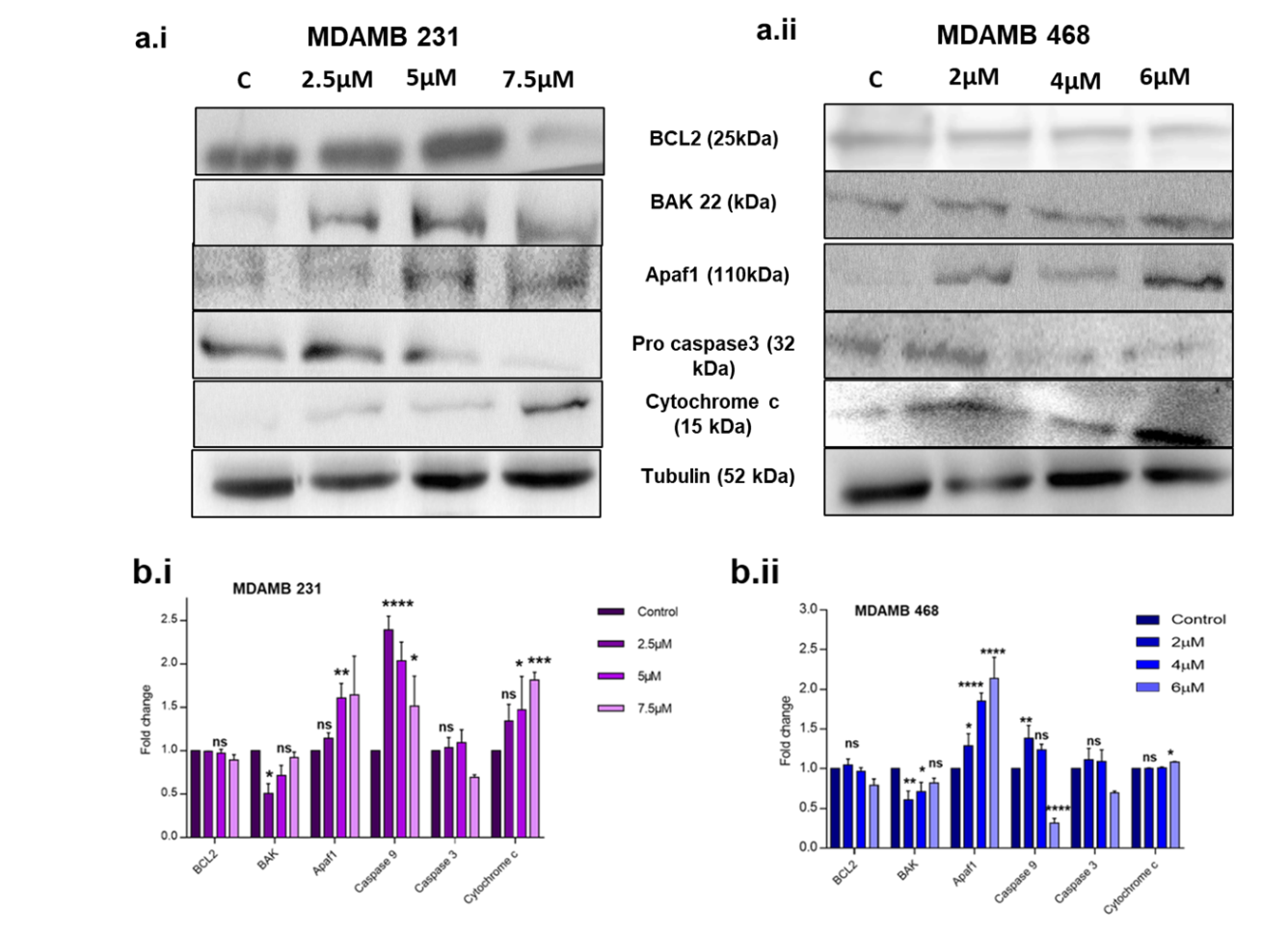


**
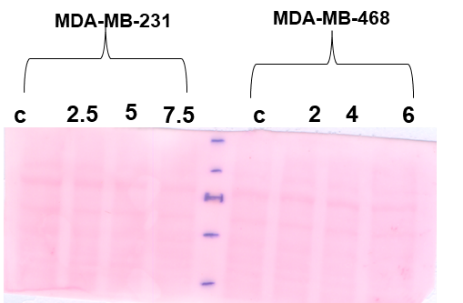
c.**

**Figure S2:** Effect of Disarib treatment on the expression of apoptotic proteins in **a.i** Expression level of apoptotic proteins following Disarib treatment in MDA-MB-231 cell line and **b.i** Quantification. bar graphs for the apoptotic proteins in MDA-MB-231 **a.ii** Expression level of apoptotic proteins following Disarib treatment in MDA-MB-468 cell line and **b.ii** Quantification. bar graphs for the apoptotic proteins in MDA-MB-468. c. Representative ponceau image of the blot. Each experiment has been repeated a minimum of two times, and band intensities were quantified and plotted as a bar graph. The graph shows mean # SEM (ns:not significant, p < 0.05 = *, p< 0.01 = **, p<0.001 = ***, p < 0.0001 = ****)


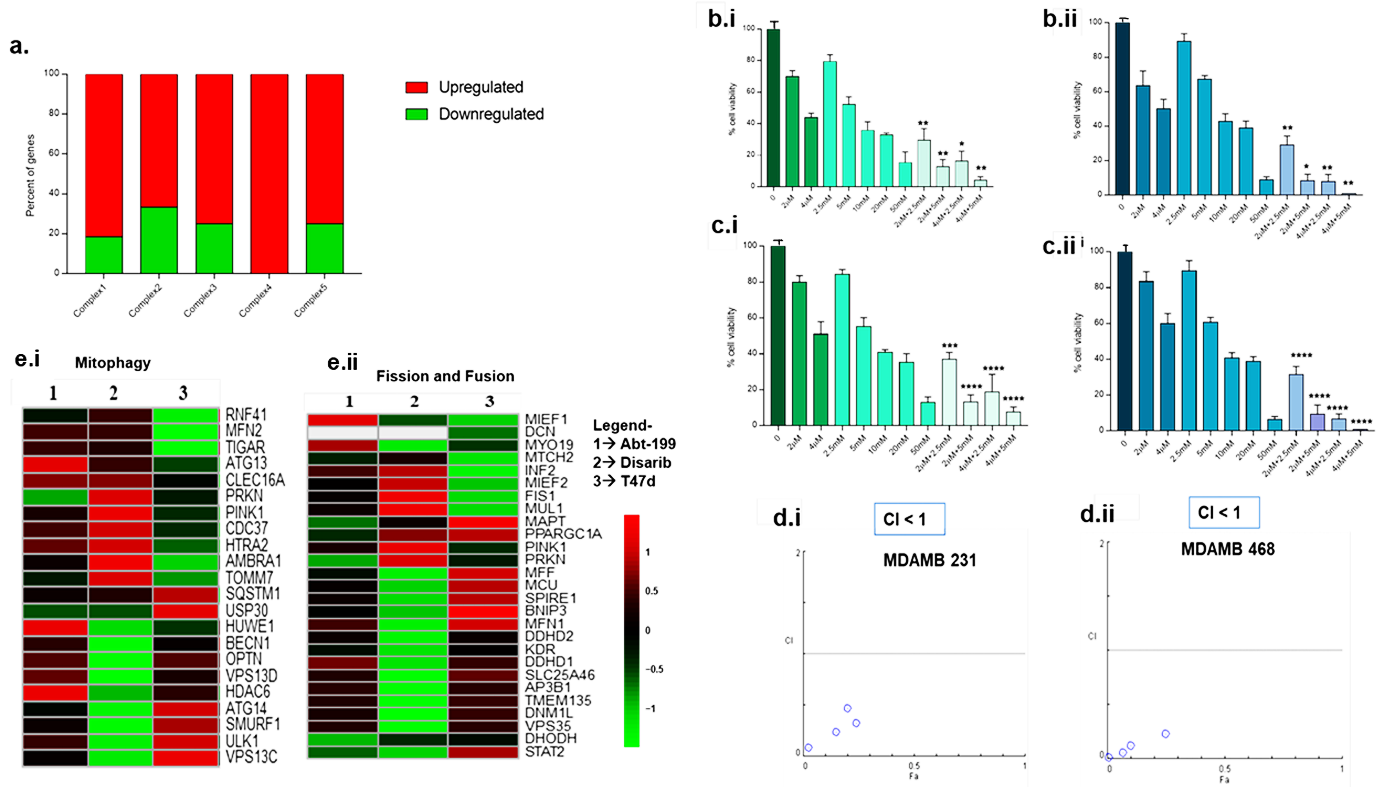


**Figure S3: a.** Percentage of up and down genes participating in OXPHOS and glycolysis in Disarib treated MDA-MB-231. Cytotoxicity assays for Disarib in combination with Metformin in **b.** MDA-MB-231 i. LDH assay **ii.** MTT assay. **c.** MDA-MB-468 **i.** LDH assay **ii.** MTT assay. The graph shows mean # SEM (ns: not significant, p < 0.05 = *, p< 0.01 = **, p<0.001 = ***, p < 0.0001 = ****). **d.** Combination index plot obtained from Chou Talalay test for Disarib and Metformin combination treatment for **i.** MDA-MB-231 **ii.** MDA-MB-468 e. Heatmaps depicting gene expression changes in Disarib and ABT199 treated MDAMB23 (Bcl2 high) and Disarib inhibitor-treated T47D (Bcl2 negligible) for i. Genes belonging to mitophagy ii. Genes from the mitochondrial fission and fusion pathway. The red colour represents upregulation, and the green colour represents downregulation.

**
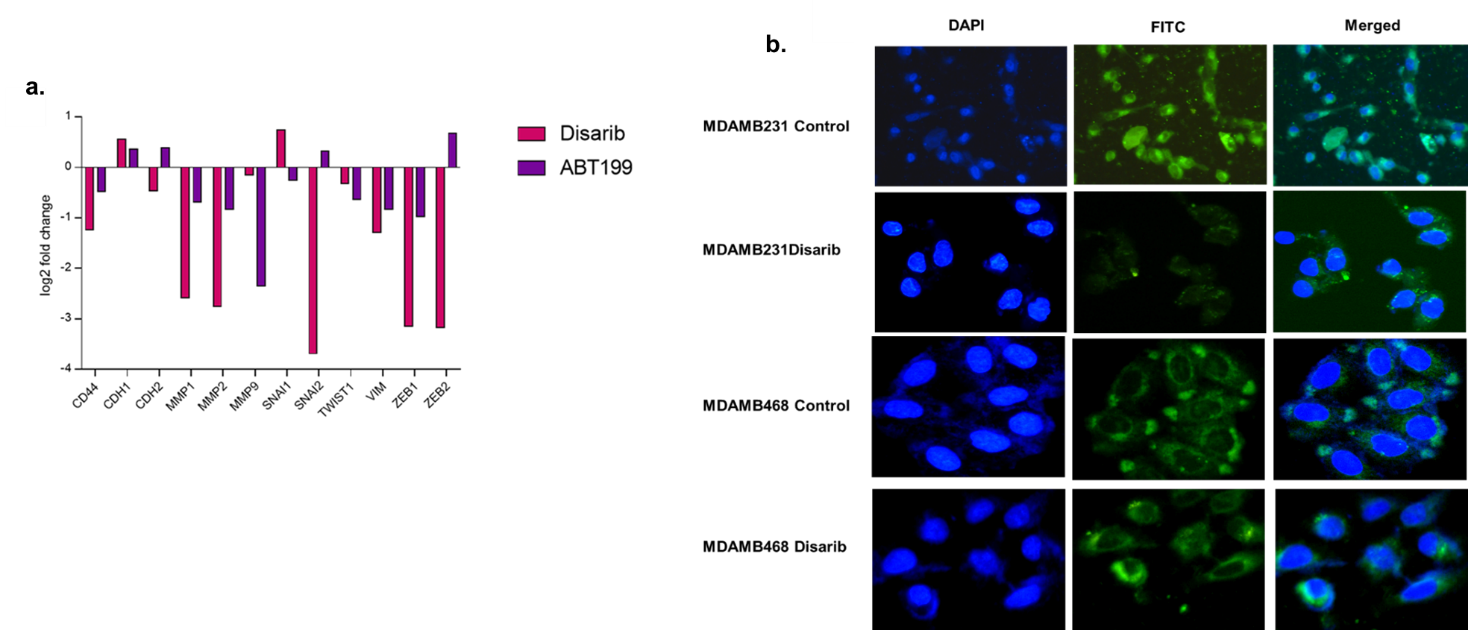
**

**Figure S4: a.** A bar graph showing log2fold change of EMT markers from the RNAseq data of Disarib and ABT-199 treated MDAMB231 b. Immunofluorescence staining of CD44 in MDA-MB-231 and MDA-MB-468 upon Disarib treatment.
