# Supplementary figures and images for: Disarib, a Specific BCL2 Inhibitor, Induces Apoptosis in Triple-Negative Breast Cancer Cells and Impedes Tumour Progression in Xenografts by Altering Mitochondria-Associated Processes
Source: Int J Mol Sci. 2024 Jun 12;25(12):6485. doi: 10.3390/ijms25126485 (PMC11203414; doi:10.3390/ijms25126485)

**Raw-original blots (Without processing)**

**
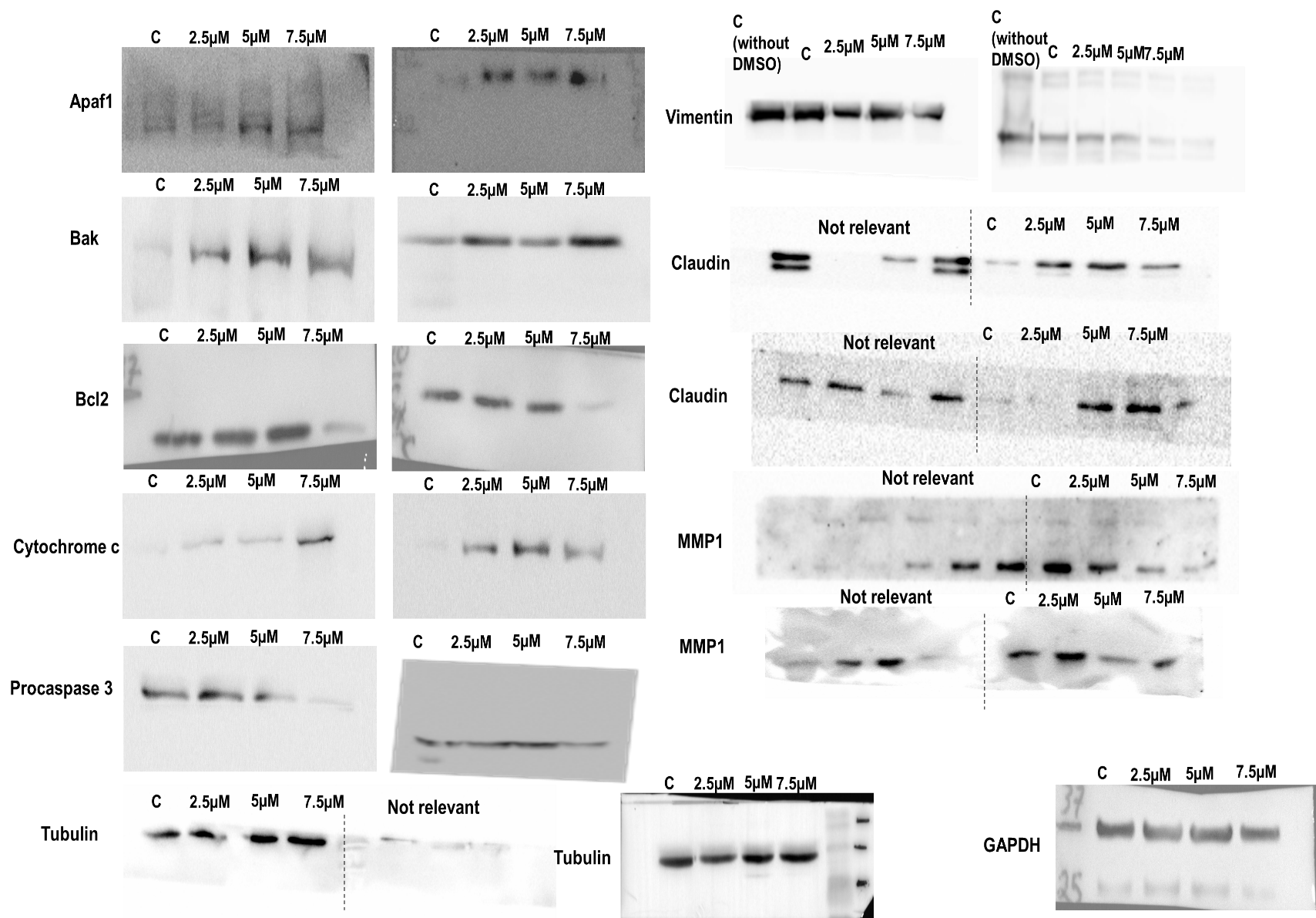
MDA-MB-231**

**
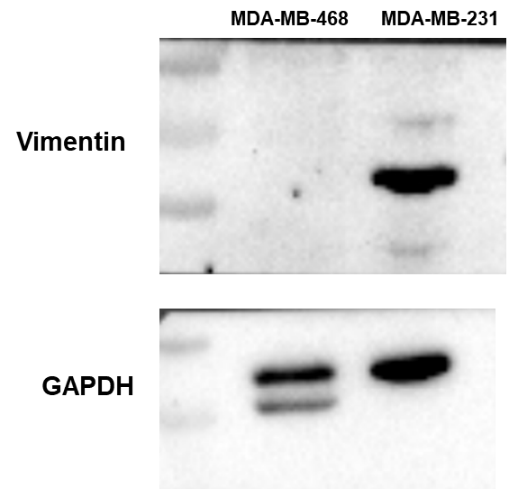
**

**
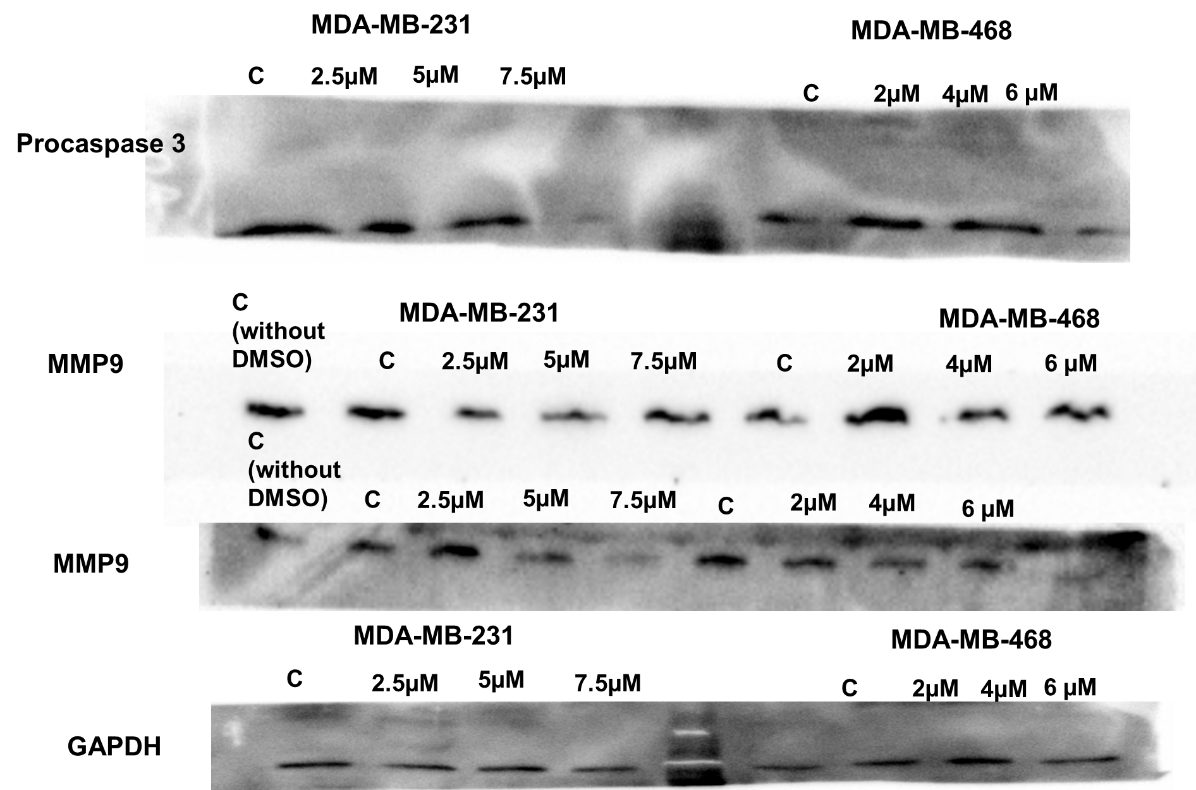
**

**
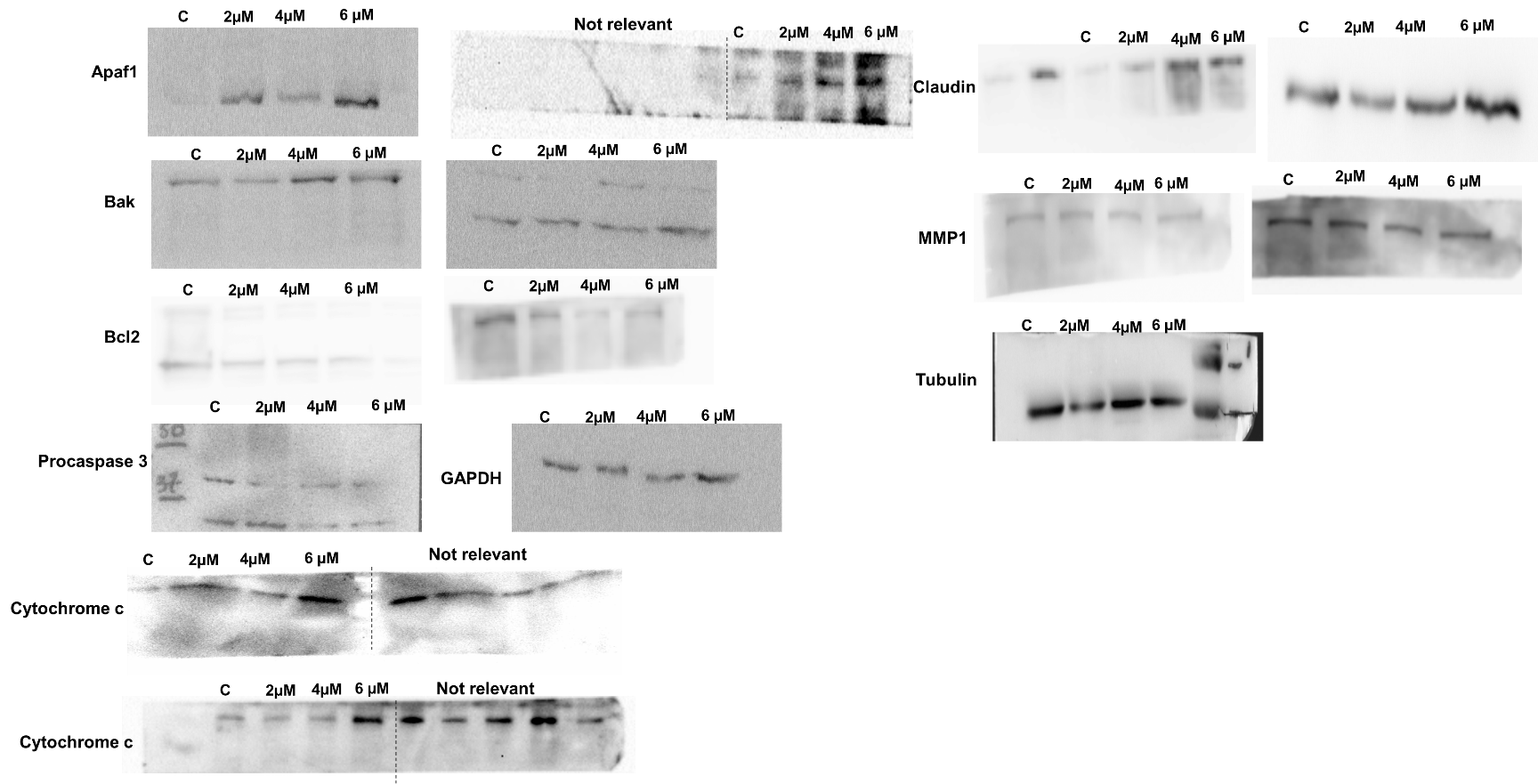
MDA-MB-468**

**
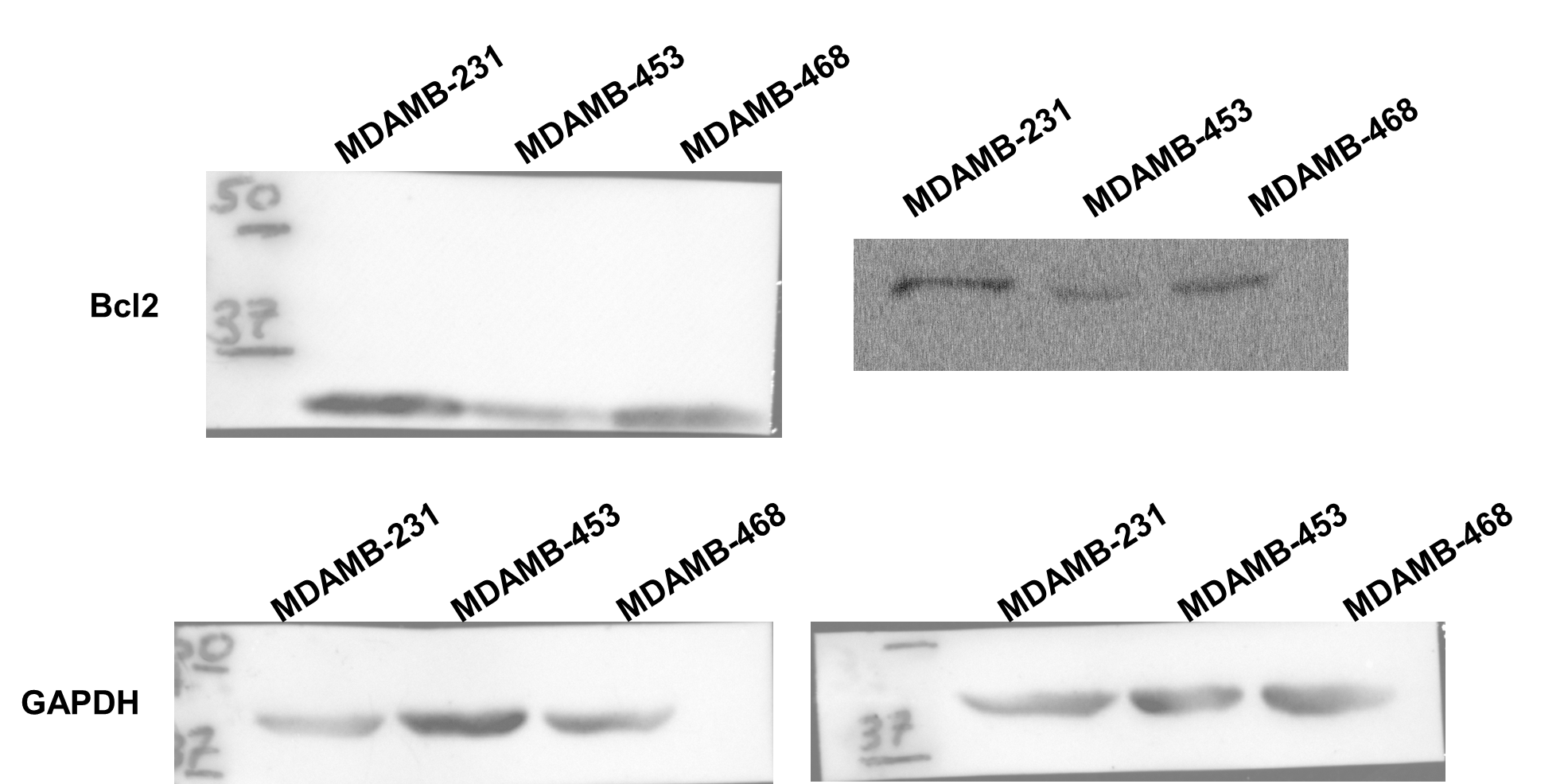
**

Supplement: Supplementary file 1 [file ijms-25-06485-s001.zip › Supplementary file2_raw files.docx]
